# Glucose-enhanced oxidative stress resistance—A protective anticipatory response that enhances the fitness of *Candida albicans* during systemic infection

**DOI:** 10.1371/journal.ppat.1011505

**Published:** 2023-07-10

**Authors:** Daniel E. Larcombe, Iryna M. Bohovych, Arnab Pradhan, Qinxi Ma, Emer Hickey, Ian Leaves, Gary Cameron, Gabriela M. Avelar, Leandro J. de Assis, Delma S. Childers, Judith M. Bain, Katherine Lagree, Aaron P. Mitchell, Mihai G. Netea, Lars P. Erwig, Neil A. R. Gow, Alistair J. P. Brown

**Affiliations:** 1 Institute of Medical Sciences, University of Aberdeen, Aberdeen, United Kingdom; 2 Medical Research Council Centre for Medical Mycology, University of Exeter, School of Biosciences, Geoffrey Pope Building, Exeter, United Kingdom; 3 Rowett Institute, School of Medicine Medical Sciences & Nutrition, University of Aberdeen, Aberdeen, United Kingdom; 4 Department of Microbiology, Biosciences Building, University of Georgia, Athens, Georgia, United States of America; 5 Department of Internal Medicine and Radboud Center for Infectious Diseases, Radboud University Medical Center, Nijmegen, Netherlands; 6 Department for Immunology & Metabolism, Life and Medical Sciences Institute, University of Bonn, Bonn, Germany; 7 Johnson-Johnson Innovation, EMEA Innovation Centre, One Chapel Place, London, United Kingdom; University of Melbourne, AUSTRALIA

## Abstract

Most microbes have developed responses that protect them against stresses relevant to their niches. Some that inhabit reasonably predictable environments have evolved anticipatory responses that protect against impending stresses that are likely to be encountered in their niches–termed “adaptive prediction”. Unlike yeasts such as *Saccharomyces cerevisiae*, *Kluyveromyces lactis* and *Yarrowia lipolytica* and other pathogenic *Candida* species we examined, the major fungal pathogen of humans, *Candida albicans*, activates an oxidative stress response following exposure to physiological glucose levels before an oxidative stress is even encountered. Why? Using competition assays with isogenic barcoded strains, we show that “glucose-enhanced oxidative stress resistance” phenotype enhances the fitness of *C*. *albicans* during neutrophil attack and during systemic infection in mice. This anticipatory response is dependent on glucose signalling rather than glucose metabolism. Our analysis of *C*. *albicans* signalling mutants reveals that the phenotype is not dependent on the sugar receptor repressor pathway, but is modulated by the glucose repression pathway and down-regulated by the cyclic AMP-protein kinase A pathway. Changes in catalase or glutathione levels do not correlate with the phenotype, but resistance to hydrogen peroxide is dependent on glucose-enhanced trehalose accumulation. The data suggest that the evolution of this anticipatory response has involved the recruitment of conserved signalling pathways and downstream cellular responses, and that this phenotype protects *C*. *albicans* from innate immune killing, thereby promoting the fitness of *C*. *albicans* in host niches.

## Author summary

In response to certain host signals, the major fungal pathogen *Candida albicans* activates anticipatory responses that protect it against impending attack by innate immune cells. These responses include immune evasion via the masking or shaving of pathogen-associated molecular patterns in response to lactate, hypoxia, iron depletion or neutral pH. These responses also include glucose-induced resistance to acute oxidative stress, which protects the fungus against killing by innate immune cells. In this study we define key signalling and cellular mechanisms that underlie “glucose-enhanced oxidative stress resistance”. This phenotype is regulated by the glucose repression and cyclic AMP-protein kinase A signalling pathways, and is dependent on the accumulation of the known stress-protectant, trehalose, in response to glucose. Glucose-enhanced oxidative stress resistance is not observed in environmental yeasts or in other pathogenic *Candida* species, including *Candida dubliniensis*, a close cousin of *C*. *albicans*. This reinforces the view that, due to the additional fitness costs they impose, the anticipatory responses displayed by a microbe reflect the environmental pressures it has recently faced in the niches in which it has evolved.

## Introduction

Anticipatory protective responses are thought to enhance the fitness of bacteria and fungi that inhabit niches that expose them to predictable environmental challenges [1]. Over evolutionary time, such microbes have “learned” to exploit one type of environmental signal to pre-emptively activate a protective response against a second environmental challenge that is likely to follow the first. In this way, an anticipatory protective response is mounted before the microbe has even encountered the second challenge, thereby enhancing their fitness and ability to survive this challenge when it arises. For example, in the context of metabolic adaptation, if the exhaustion of one carbon source is frequently associated with another becoming available, then a new metabolic programme may be induced before the substrate for its metabolism becomes available [1]. This phenomenon, which has been termed “adaptive prediction”, is thought, for example, to account for the linkage of lactose and maltose utilisation patterns in *Escherichia coli* and the development of the core environmental response in *Saccharomyces cerevisiae*. This core environmental response represents the transcriptional induction of a core set of stress genes in response to a wide range of stresses that include heat shock, osmotic and oxidative stresses and the diauxic transition [2,3] which enhances the fitness of *S*. *cerevisiae* by providing cross-protection against many of these environmental stresses [4].

Other evolutionarily divergent yeasts, such as *Schizosaccharomyces pombe* and *Candida glabrata*, display analogous core environmental responses to *S*. *cerevisiae*. However, *Candida albicans* does not display such an analogous core environmental response [5–7]. Instead, this major fungal pathogen of humans displays alternative anticipatory protective responses. These include the secretion of candidalysin and the Pra1 zincophore during hyphal development [8,9] which is thought to promote nutrient and micronutrient assimilation as the fungal hypha penetrates host tissues within the potentially nutrient-limited environment of the invasion pocket [10,11]. *C*. *albicans* also actively manages the exposure of a major pathogen-associated molecular pattern at its cell surface, β-1,3-glucan, in response to specific host signals such as ambient pH, the gut fermentation acid lactate, hypoxia and iron limitation [12–16]. By reducing β-1,3-glucan exposure at its cell surface in response to these host signals, even before the fungus encounters macrophages or neutrophils, *C*. *albicans* reduces the ability of innate immune cells to recognise it and therefore to mount antifungal responses [12,14,15]. Furthermore, in contrast to *S*. *cerevisiae*, *C*. *albicans* responds to physiological concentrations of glucose by enhancing its resistance to subsequent exposure to an acute oxidative stress [17] via mechanisms that are explored in this study. This phenomenon, termed “glucose-enhanced oxidative stress resistance”, is predicted to enhance the fitness of *C*. *albicans in vivo* by protecting against phagocytic killing using reactive oxygen species [11]. Therefore, *C*. *albicans* displays different forms of anticipatory protective response to *S*. *cerevisiae* and *S*. *pombe*.

Anticipatory protective responses come with an additional fitness cost associated with the activation of a physiological response before it is actually required [1]. Consequently, during their development over evolutionary time, the costs of anticipatory protective responses will have been weighed against the subsequent fitness benefits they provide. These anticipatory responses may arise through mutations that create new network connections between different signalling or transcriptional modules: a missense mutation that introduces a new protein kinase target site on a signalling component on another pathway could bring this pathway under the control of this protein kinase [18]. New network connections can evolve relatively rapidly. For example, when *S*. *cerevisiae* is placed under selective pressures involving the repetitive, sequential imposition of two different stresses *in vitro*, this yeast can evolve a corresponding anticipatory protective response relatively quickly, within 50 generations [19]. Equally, anticipatory responses can be lost relatively quickly if the corresponding selective pressures become temporally unlinked [1]. Therefore, anticipatory protective responses are likely to reflect an evolutionary “memory” of relatively recent selective pressures [11]. This presumably explains the significant differences between *S*. *cerevisiae*, *S*. *pombe* and *C*. *albicans* in terms of the nature of the anticipatory protective responses they display [18].

In this study we have focused on glucose-enhanced oxidative stress resistance in *C*. *albicans*. We have tested whether this anticipatory response protects this pathogenic fungus against phagocytic attack and whether it enhances fungal fitness during systemic infection. We have also explored the mechanistic basis for glucose-enhanced oxidative stress resistance and how this phenotype is regulated. In doing so we confirm the relevance of anticipatory protective responses to fungal infection.

## Results

### Glucose-enhanced oxidative stress resistance promotes the fitness of C. albicans in vivo

**Fig 1:**
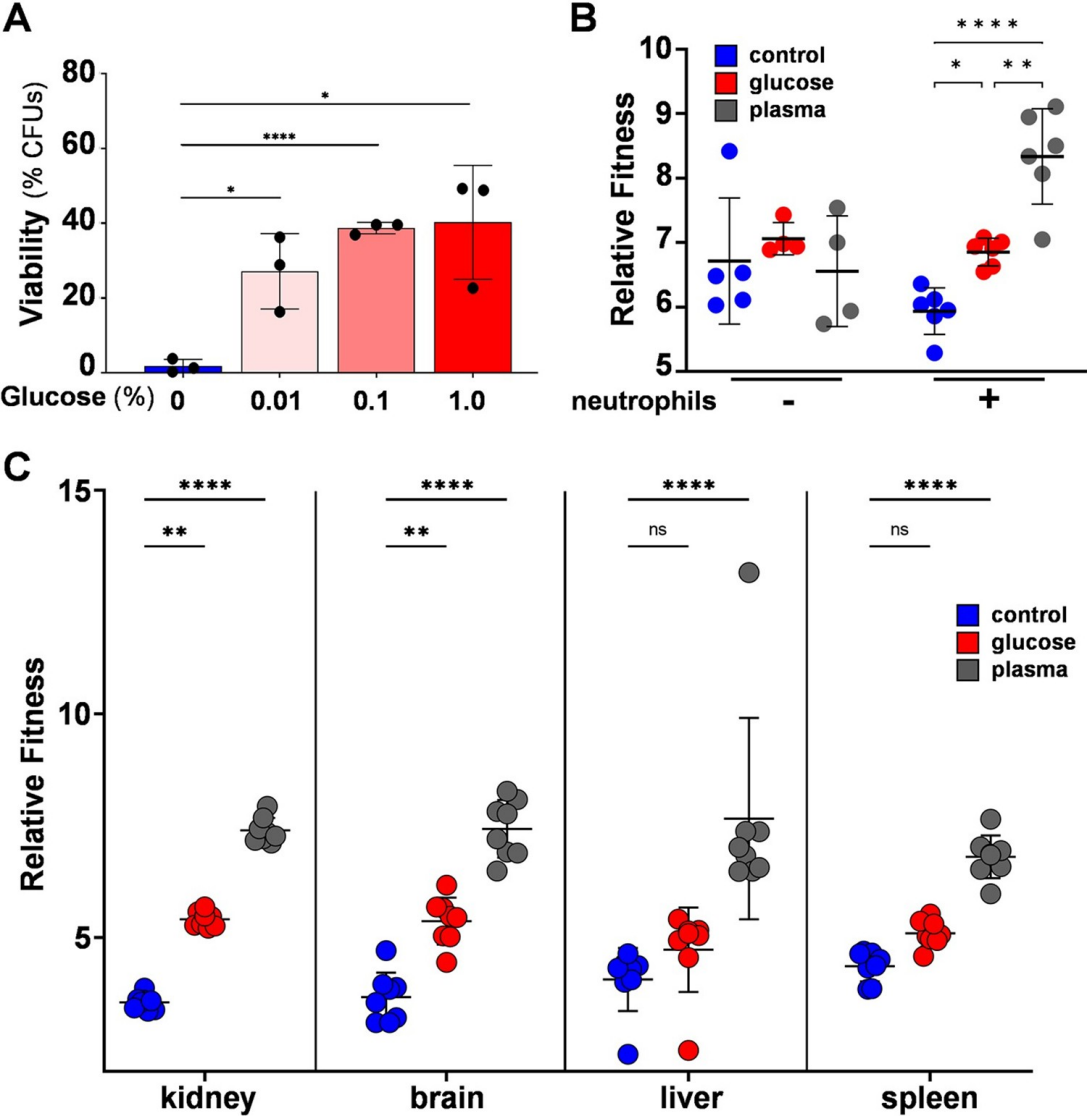
Exposure to glucose or plasma enhances the robustness of *C*. *albicans in vitro*, *ex vivo* and *in vivo*. **(A)** Glucose enhances oxidative stress resistance *in vitro*. *C*. *albicans* RM1000 (S1 Table) was grown on lactate (YPL), incubated for 1 h with glucose at the specified concentrations, and then exposed to 50 mM H_2_O_2_ for 1 h, after which cell viability was then assayed (CFUs). Each data point represents the mean of three technical replicates, statistical analyses were performed using multiple unpaired t tests, comparing all groups with the control. Means and standard deviations are shown: *, *p* ≤ 0.05; **, *p* ≤ 0.01; ***, *p* ≤ 0.001; ****, *p* ≤ 0.0001. **(B)** Pre-exposure to glucose or plasma increases the resistance of *C albicans* to PMN killing. Isogenic, wild type, barcoded *C*. *albicans* strains (S1 Table) were exposed to 0% or 1% glucose or 20% plasma for 1 h, pooled and co-incubated with human PMNs for 3 h. After plating on YPD to select for viable cells, the relative proportion of each barcode (n = 5 per condition) was assayed by barcode sequencing and normalised against the proportion in the original pool before PMNs exposure. Means and standard deviations for PNNs from 6 different healthy donors are shown. **(C)** Pre-exposure to glucose or plasma increases the fitness of *C albicans* during systemic infection in mice. Barcoded *C*. *albicans* strains (S1 Table) were exposed to 0% or 1% glucose or 20% plasma for 1 h, pooled, and injected into the tail vein of BALB/c mice. The mice were culled one day later, and fungal cells in the kidneys, liver, spleen and brain harvested for analysis. Barcode sequencing was then performed to determine the relative proportion of each strain before and after infection. Each data point represents the mean of the technical replicates (n = 4 barcodes for each condition) from a single mouse (n = 8). Means and standard deviations for the eight mice are presented. The data in (B) and (C) were analysed using two-way ANOVA with Tukey’s multiple comparisons test.

Our first objective was to test the prediction that glucose-enhanced oxidative stress resistance promotes the fitness of *C*. *albicans in vivo* [11,17]. First, we reconfirmed the phenotype by showing that *C*. *albicans* activates oxidative stress resistance in response to a wide range of glucose concentrations *in vitro*. Cells were grown on rich medium containing lactate as the main carbon source (YPL), and then exposed to glucose for one hour. The resistance of the *C*. *albicans* cells to an acute oxidate stress (50 mM hydrogen peroxide: H_2_O_2_) was then assayed. As we reported previously [17], oxidative stress resistance was significantly enhanced by exposing cells to glucose at all three concentrations tested and, in particular, to physiologically relevant glucose concentrations (Fig 1A). A concentration of 0.01% approximates to blood glucose levels [20].

Having recapitulated the phenotype, we then tested whether glucose-enhanced oxidative stress resistance promotes resistance to phagocytic attack. To achieve this, we used barcode sequencing to compare the fitness of differentially pre-adapted *C*. *albicans* cells during exposure to polymorphonuclear leukocytes (PMNs). Briefly, a set of isogenic prototrophic *C*. *albicans* strains each with a unique barcode (S1 Table) were grown in parallel on YPL. Five barcoded *C*. *albicans* strains were then exposed to 1% glucose, five strains exposed to 20% human plasma, and five strains exposed to water alone (control). After one hour, the 15 barcoded strains were quickly harvested, washed and pooled, and then co-incubated for three hours with human PMNs from six healthy volunteers. Viable *C*. *albicans* cells were rescued by plating on YPD, genomic DNA was isolated from the pooled colonies, and the relative proportion of each barcoded strain was determined by barcode sequencing. Prior glucose exposure significantly enhanced the fitness of *C*. *albicans* during phagocytic attack (Fig 1B). Interestingly, exposure to plasma afforded an even higher level of protection.

We then performed an analogous barcoding experiment to test the impact of glucose-enhanced oxidative stress resistance upon *C*. *albicans* fitness during systemic infection in mice. Once again, five barcoded *C*. *albicans* strains were exposed to 1% glucose, 20% human plasma or water alone (control) and, after one hour, the 15 barcoded strains were harvested, washed and pooled. This pool of barcoded strains was then used to infect 8 mice and these mice culled after two days. Fungal cells from the kidney, brain, liver and spleen were plated onto YPD, and the relative proportions of barcoded strains assayed by sequencing (Fig 1C). Exposure to glucose significantly increased the fitness of *C*. *albicans* cells in the kidney and brain, and similar trends were observed in the liver and spleen although these changes did not reach statistical significance. Again, the impact of plasma was greater than for glucose. Given that the differentially pre-adapted *C*. *albicans* cells will have started to re-adapt to their new surroundings following injection into mice, we reason that the observed fitness advantages were likely to have been realised early in the infection during host fungus interactions in the bloodstream. We conclude that, as predicted [11,17], glucose-enhanced oxidative stress resistance increases the ability of *C*. *albicans* to resist phagocytic attack and promotes fitness during systemic infection.

### Glucose-enhanced oxidative stress resistance is dependent on glucose signalling

**Fig 2:**
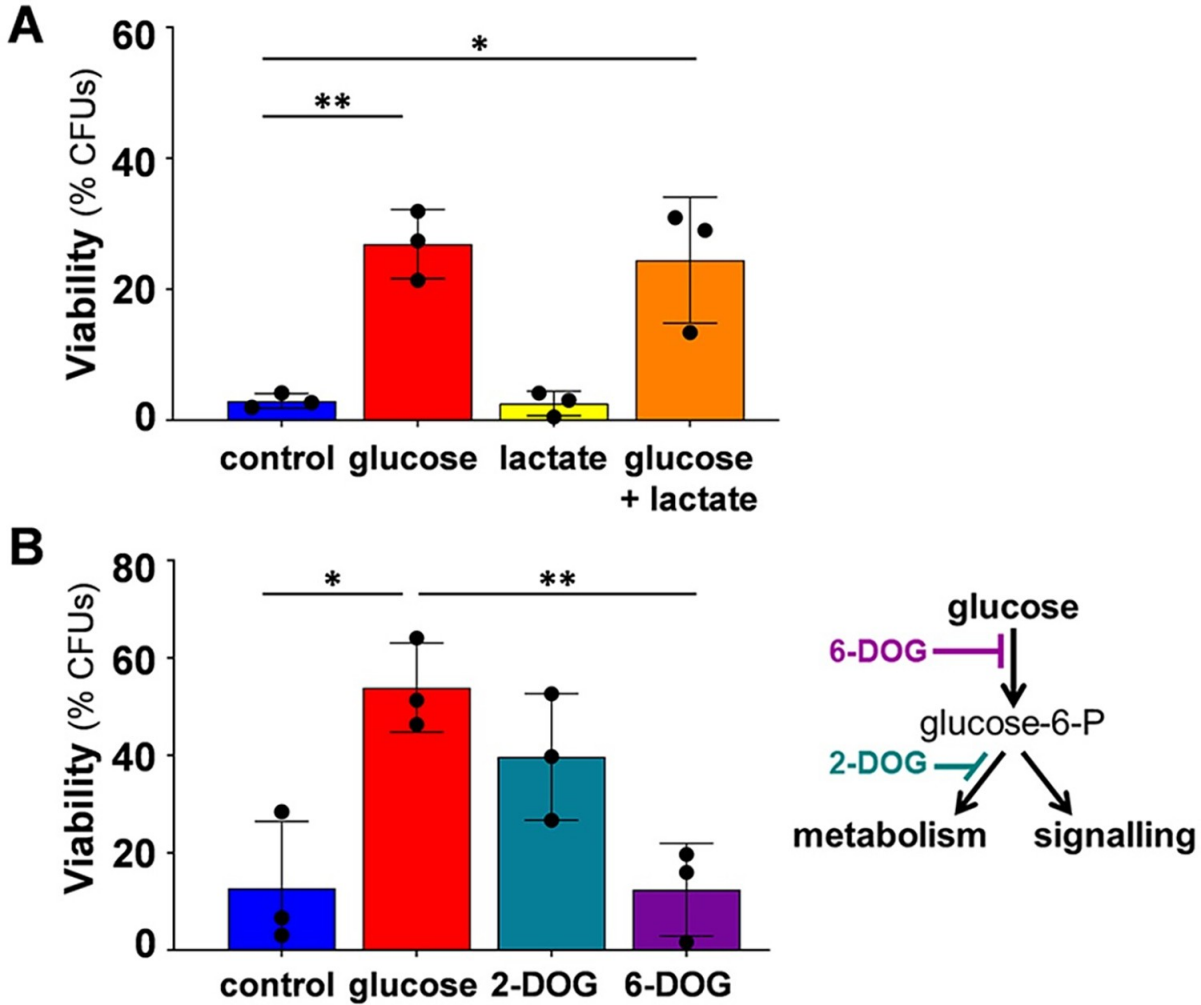
Glucose signalling is required to activate glucose-enhanced oxidative stress resistance. **(A)** Lactate does not trigger glucose-enhanced oxidative stress resistance. *C*. *albicans* SC5314 (S1 Table) was grown on citrate (YPC) and then exposed for 1 h to carrier (H_2_O), 1% glucose, 1% lactate, or a combination of both. The cells were then exposed to 25 mM H_2_O_2_ for 1 h and cell viability then assayed (CFUs). **(B)** Glucose signalling, not metabolism, triggers glucose-enhanced oxidative stress resistance. *C*. *albicans* RM1000 was grown on lactate (YPL), incubated for 1 h with 1% glucose, 2-deoxyglucose or 6-deoxyglucose, exposed to 50 mM H_2_O_2_ for 1 h, and cell viability assayed (CFUs). The data in (A) and (B) were analysed using multiple unpaired t tests, comparing all groups with the control. Means and standard deviations for triplicate experiments are presented: *, *p* ≤ 0.05; **, *p* ≤ 0.01.

The glucose-enhanced oxidative stress resistance phenotype was defined using *C*. *albicans* cells pre-grown on lactate [17]. Exposing *C*. *albicans* cells to lactate is known to trigger physiological responses such as altered stress resistance, cell wall remodelling, and β-1,3-glucan masking at the cell surface [12,21,22]. Therefore, we tested whether glucose-enhanced oxidative stress resistance is actually dependent upon exposure to lactate rather than glucose. To achieve this, *C*. *albicans* cells were pre-grown on YP containing citrate instead, and then exposed to 1% lactate, 1% glucose or a combination of both. The resistance to oxidative stress was only enhanced in response to glucose, not lactate (Fig 2A). Therefore, the glucose-enhanced oxidative stress resistance phenotype is dependent upon glucose pre-exposure, not upon lactate.

Then, using analogues of glucose, we tested whether the activation of glucose-enhanced oxidative stress resistance is dependent upon glucose metabolism or glucose signalling. 2-deoxyglucose cannot be metabolised but can be phosphorylated, and hence triggers glucose signalling [23]. In contrast, 6-deoxyglucose cannot be metabolised or phosphorylated and, consequently, cannot activate glucose signalling [24]. Significantly, glucose-enhanced oxidative stress resistance was induced by 2-deoxyglucose, but not by 6-deoxyglucose (Fig 2B). This indicated that the phenotype is dependent upon glucose signalling.

### The Sugar Receptor Repressor pathway is not required for glucose-enhanced oxidative stress resistance

Three main pathways are thought to mediate glucose signalling in *C*. *albicans*: the Sugar Receptor Repressor (SRR) pathway, the glucose repression pathway, and the cyclic AMP-protein kinase A (cAMP-PKA) pathway [25]. First, we examined the role of the SRR pathway in triggering glucose-enhanced oxidative stress resistance.

**Fig 3:**
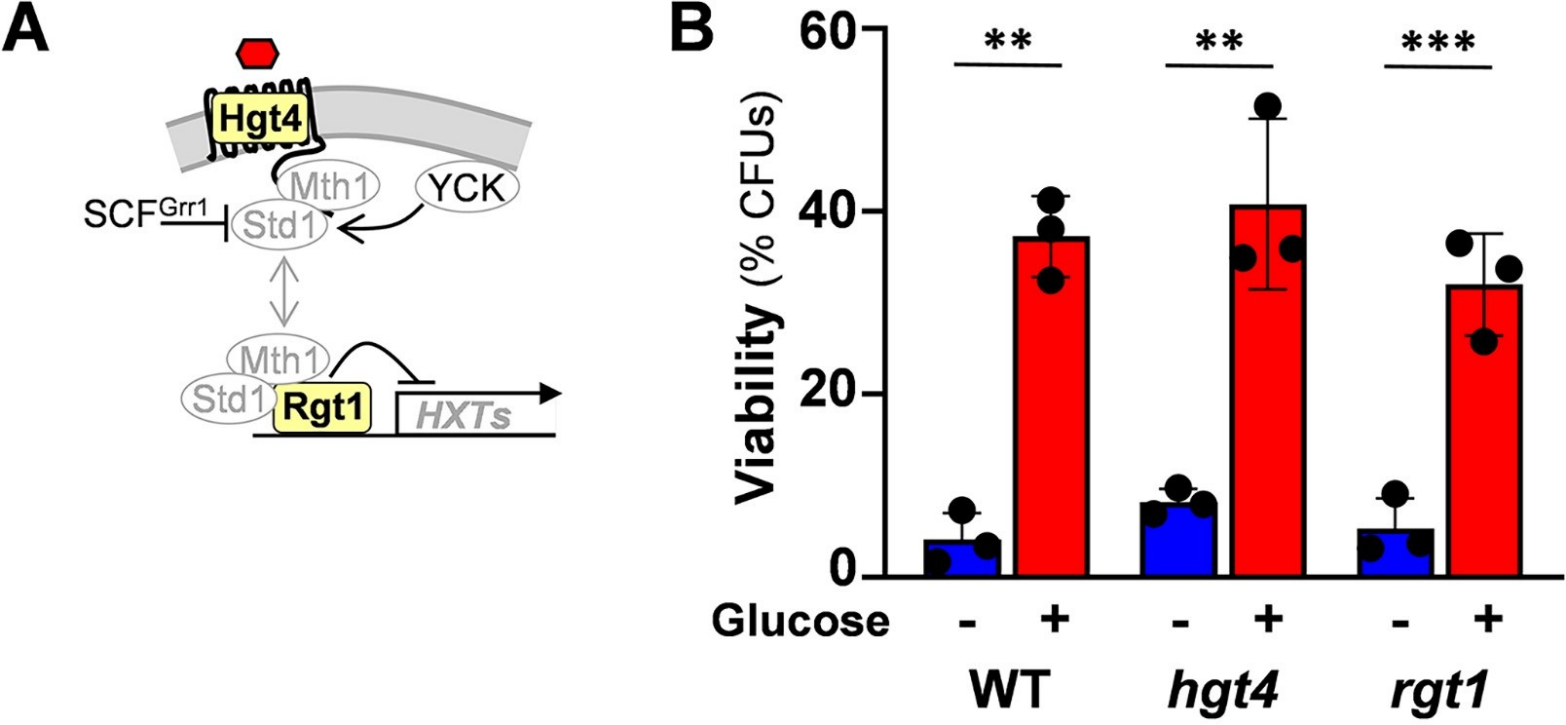
The sugar receptor repressor pathway is not required for glucose-enhanced oxidative stress resistance. **(A)** Diagrammatic representation of the sugar receptor repressor (SRR) pathway highlighting the components analysed: Hgt4, Rgt1, in bold. In the absence of glucose Std1 and Mth1 mediate transcriptional repression of glucose transporter genes by Rgt1 (see text). In the presence of glucose, the sugar is detected by the sensor Hgt4. This leads to the activation of YCK, which promotes the targeting of Std1 and Mth1 by the SCF^Grr1^ complex for ubiquitin-mediated degradation, and the derepression of sugar importer genes (see text). **(B)** *C*. *albicans* cells were cultured on lactate (YPL), incubated for 1 h with 1% glucose, exposed to 50 mM H_2_O_2_ for 1 h, and cell viability assayed (CFUs): *C*. *albicans* BWP17, wild type, WT; *hgt4*; *rgt1* (S1 Table). The data were analysed using two-way ANOVA, comparing each test (plus glucose) to the corresponding control (minus glucose). Means and standard deviations for triplicate experiments are presented: *, *p* ≤ 0.05; **, *p* ≤ 0.01; ***, *p* ≤ 0.001.

Hgt4 and Rgt1 lie on the SRR pathway in *C*. *albicans*, mediating the derepression of sugar importers in response to glucose [26,27] (Fig 3A). By analogy with *S*. *cerevisiae*, in the absence of glucose, the co-repressors Std1 and Mth1 promote transcriptional repression by the Zn(II)_2_Cys_6_ cluster DNA-binding protein, Rgt1 [28]. Upon exposure to glucose, the sugar is detected by the sensor Hgt4, which activates yeast protein kinase C (YCK) leading to the ubiquitination of Std1 and Mth1 by the Skp1-Cdc53-F-box protein (SCF^Grr1^) complex, and their degradation via the proteasome [28]. Ultimately, this releases sugar importer genes from their transcriptional repression by Rgt1 in response to glucose (Fig 3A).

We tested whether the inactivation of Hgt4 or Rgt1 in *C*. *albicans* blocks glucose-enhanced oxidative stress resistance. *C*. *albicans hgt4*Δ and *rgt1*Δ null mutants were grown on YPL, exposed to 1% glucose for one hour, and then their resistance to oxidative stress assayed. Both *hgt4*Δ and *rgt1*Δ cells retained the phenotype (Fig 3B), indicating that the SRR signalling pathway is not required for glucose-enhanced oxidative stress resistance.

### Components of the glucose repression pathway modulate oxidative stress resistance

**Fig 4:**
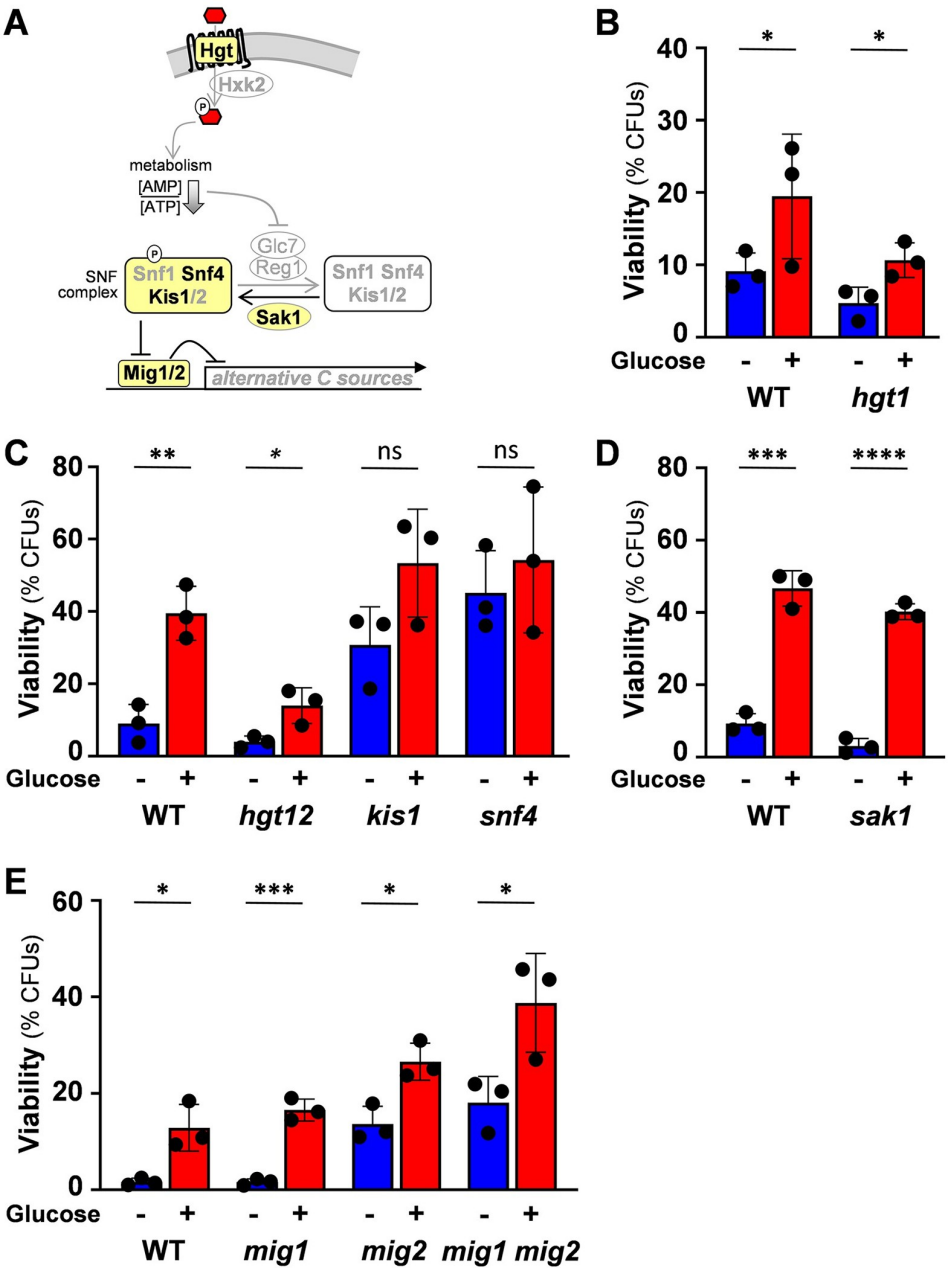
Components of the glucose repression pathway influence glucose-enhanced oxidative stress resistance. **(A)** Diagrammatic representation of the glucose repression pathway highlighting the analysed components: Hgt1, Hgt12, Sak1, Snf4, Kis1, Mig1, Mig2, in bold. In the absence of glucose, Sak1 activates Snf1, thereby permitting growth of *C*. *albicans* on alternative carbon sources (see text). Following glucose import via hexose transporters (e.g. Hgt1, Hgt12) the catabolised thereby increasing the energy balance (i.e. reducing the [AMP]/[ATP] ratio). This inactivates the Snf1 complex (containing Snf1, Kis1/2, Snf4) by dephosphorylation of Snf1 via the Glc7/Reg1 phosphatase. This leads to Mig1/2 activation and the repression of genes involved in the utilisation of alternative carbon sources (see text). To assay the contributions of pathway components to glucose-enhanced oxidative stress resistance, *C*. *albicans* strains (S1 Table) were cultured on lactate (YPL), incubated for 1 h with 1% glucose, exposed to 50 mM H_2_O_2_ for 1 h, and their cell viability assayed (CFUs). **(B)** Hgt1 is not required for the response: *C*. *albicans* SC5314, wild type, WT; *hgt1*. **(C)** Snf4 inactivation derepresses glucose-enhanced oxidative stress resistance: *C*. *albicans* DAY286, wild type, WT; *hgt12*; *kis1*; *snf4*. **(D)** Sak1 is not required for the response: *C*. *albicans* SC5314, wild type, WT; *sak1*. **(E)** Inactivation of Mig2 derepresses glucose-enhanced oxidative stress resistance: *C*. *albicans* CW542, wild type, WT; *mig1*; *mig2*; *mig1 mig2*. The data in (B), (C), (D) and (E) were analysed using two-way ANOVA, comparing each test (plus glucose) to the corresponding control (minus glucose). Means and standard deviations for triplicate experiments are presented: *, *p* ≤ 0.05; **, *p* ≤ 0.01; ***, *p* ≤ 0.001; ****, *p* ≤ 0.0001.

The evolutionarily conserved AMP-activated serine/threonine kinase, Snf1, lies at the heart of the glucose repression pathway [25,28,29] (Fig 4A). Snf1 was thought to be essential for viability in *C*. *albicans* [30], unlike in *S*. *cerevisiae* [31,32], but recently the generation of viable, albeit slow growing *C*. *albicans snf1*Δ null mutants was reported [33]. The upstream kinase, Sak1, phosphorylates and activates Snf1, thereby permitting growth of *C*. *albicans* on alternative carbon sources [34]. Following glucose import via hexose transporters such as Hgt1 and Hgt12, the glucose is phosphorylated by hexokinase (Hxk2) which is then catabolised via glycolysis to increase intracellular ATP levels. By analogy with *S*. *cerevisiae* [28], this is thought to inactivate the Snf1 complex by dephosphorylation of the α-subunit, Snf1, via the Glc7/Reg1 phosphatase. In *C*. *albicans*, the heterotrimeric Snf1 complex includes an β-subunit (Kis1 or Kis2) and a γ-subunit (Snf4) [35,36]. Snf1 inactivation allows activation of the Mig1 and Mig2 transcriptional repressors, which then leads to the repression of genes involved in the assimilation of alternative carbon sources [37]. In this way, glucose exposure triggers the transcriptional repression of gluconeogenic and glyoxylate cycle genes, for example [17,37].

To examine the role of the glucose repression pathway in activating glucose-enhanced oxidative stress resistance, we first tested the impact of the hexose transporters Hgt1 and Hgt12. The *HGT1* gene is expressed at physiologically relevant concentrations of glucose [17] and is regulated by Mig1 and Mig2, which lie downstream in the glucose repression pathway [37,38]. *HGT12* is induced in response to physiological glucose concentrations and during phagocytosis [27,39,40]. However, both *C*. *albicans hgt1*Δ and *hgt12*Δ mutants displayed glucose-enhanced oxidative stress resistance (Fig 4B & 4C), indicating that neither of these transporters is essential for this phenotype. Given likely functional redundancy within the *HGT* gene family, this does not exclude the possibility that they might contribute to the phenotype.

Next, we examined whether Sak1 is required for glucose-enhanced oxidative stress resistance. *C*. *albicans sak1*Δ cells retained the phenotype (Fig 4D), indicating that Snf1 activation by Sak1 is not required. We then turned to subunits of the Snf1 complex. The phenotype was not lost in *C*. *albicans kis1*Δ cells (Fig 4C), which was consistent with the existence of functional redundancy between the β-subunits Kis1 and Kis2 during growth on alternative carbon sources [35]. Nevertheless, *kis1*Δ cells displayed elevated oxidative stress resistance irrespective of the presence of glucose (Fig 4C). Interestingly, the inactivation of the γ-subunit, Snf4, resulted in derepression of glucose-enhanced oxidative stress resistance (Fig 4C). Finally, we examined the transcriptional repressors Mig1 and Mig2, which lie on the glucose repression pathway [37] (Fig 4A). Like *kis1*Δ and *snf4*Δ cells (Fig 4C), the *C*. *albicans mig2*Δ and *mig1*Δ *mig2*Δ mutants, but not the *mig1*Δ mutant, displayed elevated oxidative stress resistance in the absence of glucose (Fig 4E), suggesting partial derepression of the glucose-enhanced oxidative stress resistance phenotype by Mig2 inactivation. Taken together, these data are consistent with the idea that the glucose repression pathway down-regulates glucose-enhanced oxidative stress resistance.

### cAMP-PKA signalling down-regulates glucose-enhanced oxidative stress resistance

**Fig 5:**
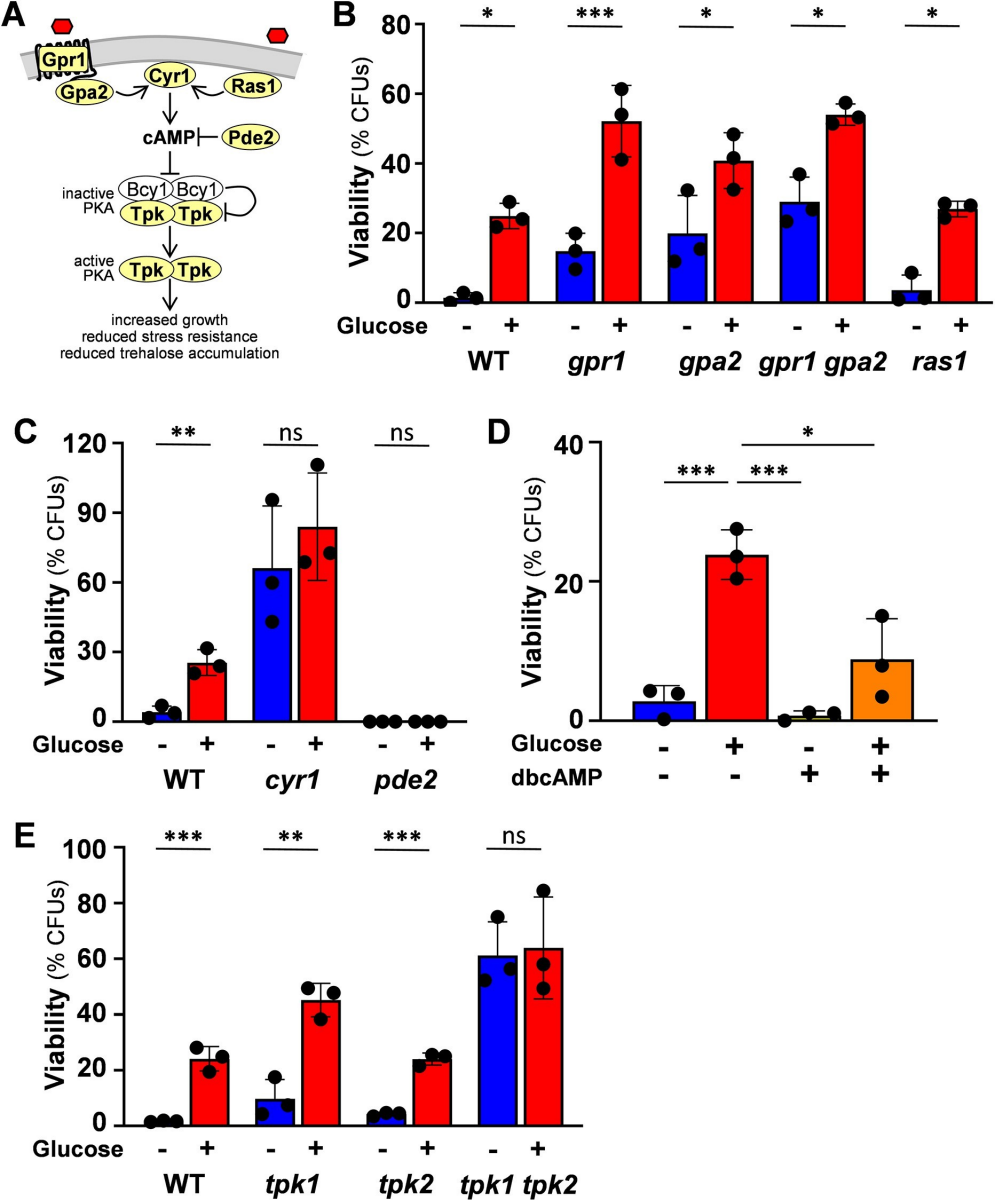
cAMP-protein kinase A signalling down-regulates glucose-enhanced oxidative stress resistance. **(A)** Diagrammatic representation of the cAMP-protein kinase A (PKA) pathway highlighting those components analysed: Ras1, Gpr1, Gpa2, Cyr1, Pde2, cAMP, Tpk1, Tpk2 (Tpk), in bold. Cyr1 can be activated by Ras1 or by Gpr1-Gpa2. Mote that Gpr1 responds to glucose in *S*. *cerevisiae*, but to methionine or lactate in *C*. *albicans* (see text). Cyr1 catalyses the production of cAMP, which is catabolised by Pde2. cAMP accumulation leads to the dissociation of Bcy1 from the protein kinase A (PKA) subunits Tpk1 and Tpk2 (Tpk). PKA then regulates its downstream targets to enhance growth rate, and down-regulate trehalose accumulation and stress resistance (see text). Components of the cAMP-PKA pathway were examined by culturing *C*. *albicans* strains (S1 Table) on lactate (YPL), incubating them for 1 h with 1% glucose, exposing the cells to 50 mM H_2_O_2_ for 1 h, and measuring their viability (CFUs). **(B)** Neither Gpr1/Gpa2 nor Ras1 are required for the response: *C*. *albicans* CAI4, wild type, WT; *gpr1*; *gpa2*; *gpr1 gpa2*; *ras1*. **(C)** Inactivation of Pde2 inhibits the response whereas Cyr1 inactivation derepresses glucose-enhanced oxidative stress resistance: *C*. *albicans* CAI4, wild type, WT; *pde2*; *cyr1*. **(D)** The cAMP analogue, dibutyryl-cyclic AMP (dbcAMP), inhibits glucose-enhanced oxidative stress resistance. *C*. *albicans* RM1000 cells were grown on lactate (YPL) and then incubated for 1 h with 1% glucose, dbcAMP, or a combination of both. The cells were then exposed to 50 mM H_2_O_2_ for 1 h, and their viability measured (CFUs). **(E)** Inactivation of PKA derepresses glucose-enhanced oxidative stress resistance: *C*. *albicans* SN152HLA, wild type, WT; *tpk1*; *tpk2*; *tpk1*, *tpk2*. In (B), (C) and (E), the data were analysed using two-way ANOVA, comparing each test (plus glucose) to the corresponding control (minus glucose). For (D), multiple comparisons were performed using one-way ANOVA. Means and standard deviations for triplicate experiments are presented: *, *p* ≤ 0.05; **, *p* ≤ 0.01; ***, *p* ≤ 0.001; ****, *p* ≤ 0.0001.

The third main glucose signalling pathway in *C*. *albicans* is the cAMP-PKA pathway, the induction of which is dependent on the activation of adenylyl cyclase (Cyr1) [25,41] (Fig 5A). Cyr1 can be activated by the Ras1 GTPase or by the G-protein coupled receptor Gpr1 and its G-protein α-subunit Gpa2 [42–45]. In *S*. *cerevisiae*, Gpr1 responds to glucose to activate cAMP-PKA signalling [46], whereas in *C*. *albicans* Gpr1 appears to respond to small molecules such as methionine or lactate [12,43,47]. The activation of Cyr1 leads to the accumulation of the second messenger cAMP. The phosphodiesterase Pde2 down-regulates cAMP-PKA signalling by converting cAMP to AMP [48,49]. When intracellular concentrations of cAMP increase, this second messenger interacts with Bcy1 to release its inhibition of the catalytic subunits of PKA (Tpk1, Tpk2), thereby allowing this protein kinase to modulate its downstream targets [50–53]. This leads to decreased trehalose accumulation, reduced resistance to environmental stresses, and increased growth rates [53–55] (Fig 5A).

First, we examined the roles of Ras1, Gpr1 and Gpa2 in glucose-enhanced oxidative stress resistance. This phenotype was retained by *C*. *albicans ras1*Δ, *gpr1*Δ and *gpa2*Δ single mutants and a *gpr1*Δ *gpa2*Δ double mutant (Fig 5B), indicating that none of these upstream components in the pathway are required to activate glucose-enhanced oxidative stress resistance. However, as stated above, Gpr1 and Gpa2 do not appear to mediate glucose responses in *C*. *albicans* [12,43,47]. Interestingly, the phenotype was lost in *C*. *albicans cyr1*Δ and *pde2*Δ mutants (Fig 5C). *Cyr1*Δ cells displayed constitutively high levels of oxidative stress resistance, whereas *pde2*Δ cells were extremely sensitive to this stress in the presence or absence of glucose. In addition, treatment with the permeable cAMP analogue dibutyryl cyclic AMP (dbcAMP) partially repressed the phenotype in wild type *C*. *albicans* cells (Fig 5D), indicating that cAMP levels modulate glucose-enhanced oxidative stress resistance. The glucose-enhanced oxidative stress resistance phenotype was retained by *C*. *albicans tpk1*Δ and *tpk2*Δ single mutants, but was lost in the *tpk1*Δ *tpk2*Δ double mutant, which displayed constitutive oxidative stress resistance (Fig 5E). This indicated that PKA inhibits the response. Taken together, the data clearly show that cAMP-PKA signalling down-regulates glucose-enhanced oxidative stress resistance in *C*. *albicans*.

### Changes in catalase do not mediate glucose-enhanced oxidative stress resistance

Having examined the role of glucose signalling pathways, we then explored which downstream effectors might mediate glucose-enhanced oxidative stress resistance.

**Fig 6:**
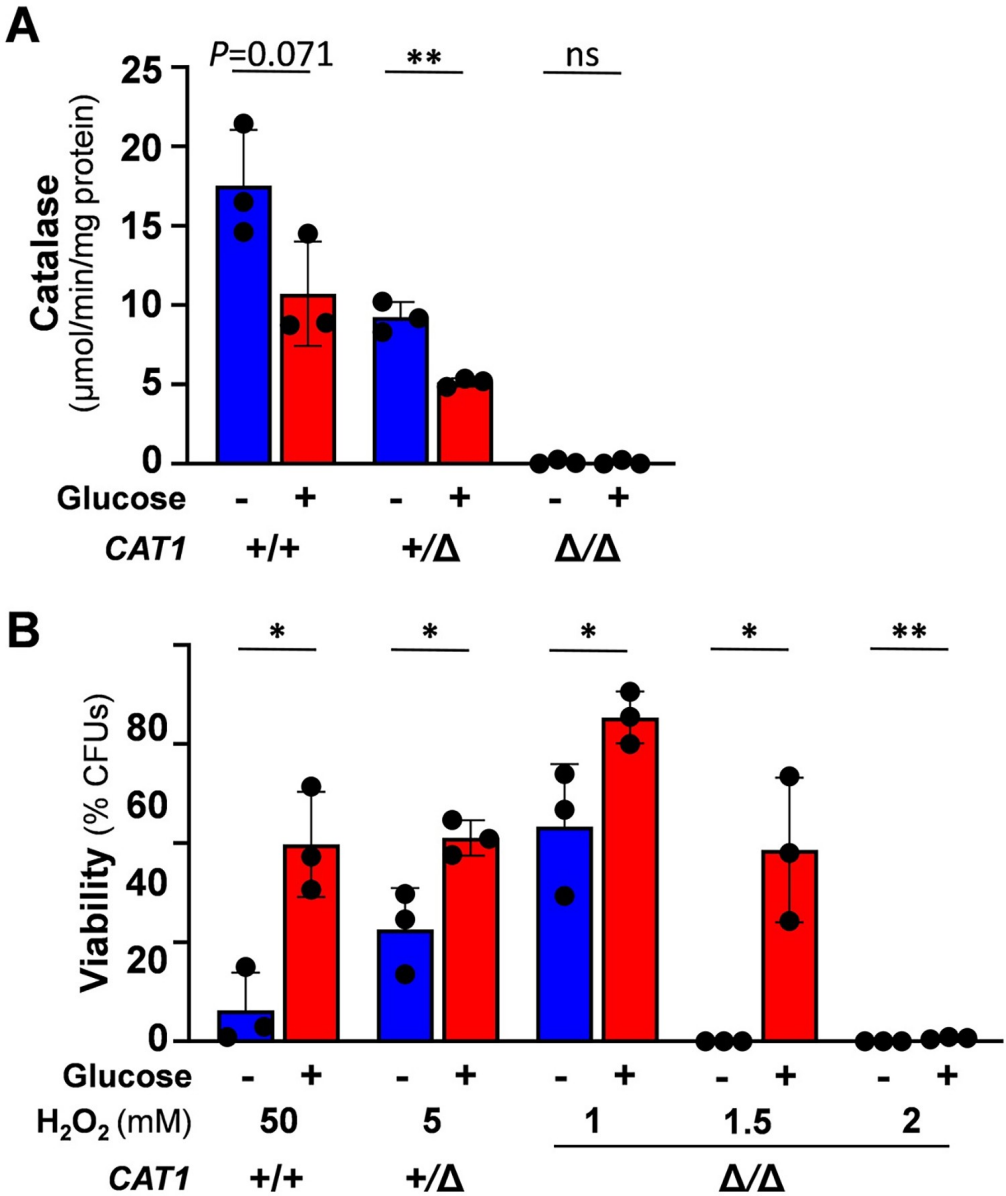
Catalase promotes oxidative stress resistance but is not required for glucose-enhanced oxidative stress resistance. **(A)** Catalase levels decrease in *C*. *albicans* in response to glucose. Catalase levels were assayed in *C*. *albicans* cells after growth on lactate (YPL) and then incubation with 0 or 1% glucose for 1 h. **(B)** Inactivating catalase does not block glucose-enhanced oxidative stress resistance. *C*. *albicans* strains were on lactate (YPL), incubated with 0 or 1% glucose for 1 h, then exposed for 1 h to H_2_O_2_ at the specified concentration, and cell viability measured (CFUs). *C*. *albicans* strains: RM1000, wild type, WT; Ca1862, *CTA1/cta1* heterozygote; Ca1864, *cta1/cta1* homozygote (S1 Table). The data in (A) and (B) were analysed using two-way ANOVA, comparing each test (plus glucose) to the corresponding control (minus glucose). Means and standard deviations for triplicate experiments are presented: *, *p* ≤ 0.05; **, *p* ≤ 0.01; ***, *p* ≤ 0.001.

Catalase catalyses the detoxification of hydrogen peroxide and is essential for resistance to this oxidative stress in *C*. *albicans* [56,57]. Therefore, we tested whether catalase levels become elevated in response to glucose. The opposite was observed: catalase activity declined in wild type cells following glucose addition (Fig 6A), which was consistent with the downregulation of *CAT1* transcript levels in response to glucose [17]. Also, enhanced oxidative stress resistance was observed in response to glucose in a homozygous *C*. *albicans cat1*Δ*/cat1*Δ mutant, despite these cells being much more sensitive to oxidative stress than wild type *CAT1/CAT1* or heterozygous *CAT1/cat1*Δ cells (Fig 6B). We conclude that changes in catalase levels do not mediate glucose-enhanced oxidative stress resistance.

### Glucose-enhanced oxidative stress resistance is not mediated by enhanced glutathione synthesis

**Fig 7:**
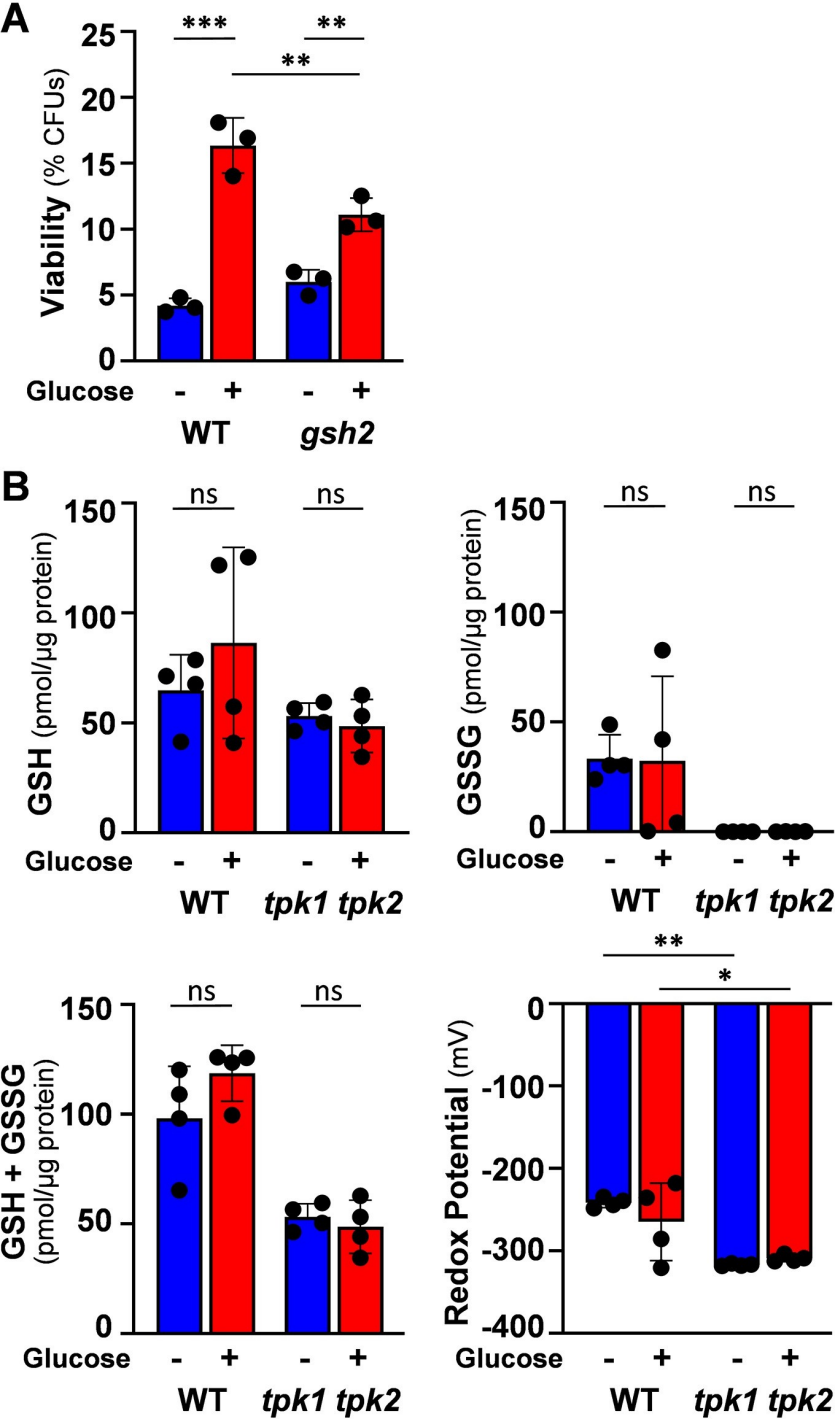
Glucose-enhanced oxidative stress resistance does not correlate with changes in glutathione concentration. **(A)** Deleting Glutathione synthetase does not block glucose-enhanced oxidative stress resistance. *C*. *albicans* strains were grown on lactate (YPL), incubated with 0 or 1% glucose for 1 h, exposed for 1 h to H_2_O_2_ at the specified concentrations, and cell viability measured (CFUs). *C*. *albicans* strains: Ca1647, RM1000+CIp20, wild type, WT; Ca2015, *gsh2* (S1 Table). **(B)** Glutathione levels in wild type and PKA-defective *C*. *albicans* cells. Reduced (GSH) and oxidised (GSSG) glutathione levels were measured in cells grown on lactate and then exposed to 0 or 1% glucose for 1 h. Panels representing GSH, GSSG, total glutathione (GSH + GSSG) and redox potential are shown. *C*. *albicans* strains: SN152HLA, wild type, WT; *tpk1 tpk2* (S1 Table). All data were analysed using two-way ANOVA, comparing each test (plus glucose) to the corresponding control (minus glucose). Means and standard deviations for triplicate experiments are presented: *, *p* ≤ 0.05; **, *p* ≤ 0.01; ***, *p* ≤ 0.001; ****, *p* ≤ 0.0001.

As the most abundant non-protein thiol in eukaryotic cells, glutathione plays a key role in maintaining redox homeostasis and the prevention of oxidative damage [58,59]. Therefore, we tested whether perturbing glutathione synthesis affects glucose-enhanced oxidative stress resistance. Glutathione synthetase (Gsh2) catalyses the last step in glutathione synthesis [60], and *C*. *albicans gsh2*Δ cells display sensitivity to oxidative stress [61]. However, *gsh2*Δ cells still displayed enhanced oxidative stress resistance in response to glucose (Fig 7A), suggesting that glutathione synthesis is not essential for this phenotype.

We then measured the levels of the reduced (GSH) and oxidised (GSSG) forms of glutathione in wild type cells before and after glucose exposure. We also examined the *tpk1*Δ *tpk2*Δ double because cAMP-PKA signalling regulates glucose-enhanced oxidative stress resistance (Fig 5). GSH levels were similar in both strains, but GSSG levels were dramatically reduced in the *tpk1*Δ *tpk2*Δ mutant (Fig 7B). This was consistent with the high levels of oxidative stress resistance displayed by this mutant (Fig 5E). Interestingly, GSH and GSSG levels did not change significantly in response to glucose in wild type or *tpk1*Δ *tpk2*Δ cells, and consequently their redox potential remained unaffected (Fig 7B). Taken together, the data indicate that glucose-enhanced oxidative stress resistance is not mediated by enhanced glutathione synthesis.

### Trehalose accumulation promotes glucose-enhanced oxidative stress resistance

Trehalose is a well-known stress protectant [62,63] that appears to act in part by promoting autophagy [64]. Trehalose is particularly important for oxidative stress resistance in *C*. *albicans* [65], and trehalose accumulation has been shown to provide protection against phagocytic killing [66]. Intracellular trehalose levels increase in response to acute peroxide stress in *C*. *albicans* as well as during late exponential growth. Furthermore, the levels of *TPS* transcripts encoding trehalose biosynthetic enzymes increase in *C*. *albicans* following glucose exposure [17]. Therefore, trehalose accumulation was a strong candidate for the downstream factor that mediates glucose-enhanced oxidative stress resistance.

**Fig 8:**
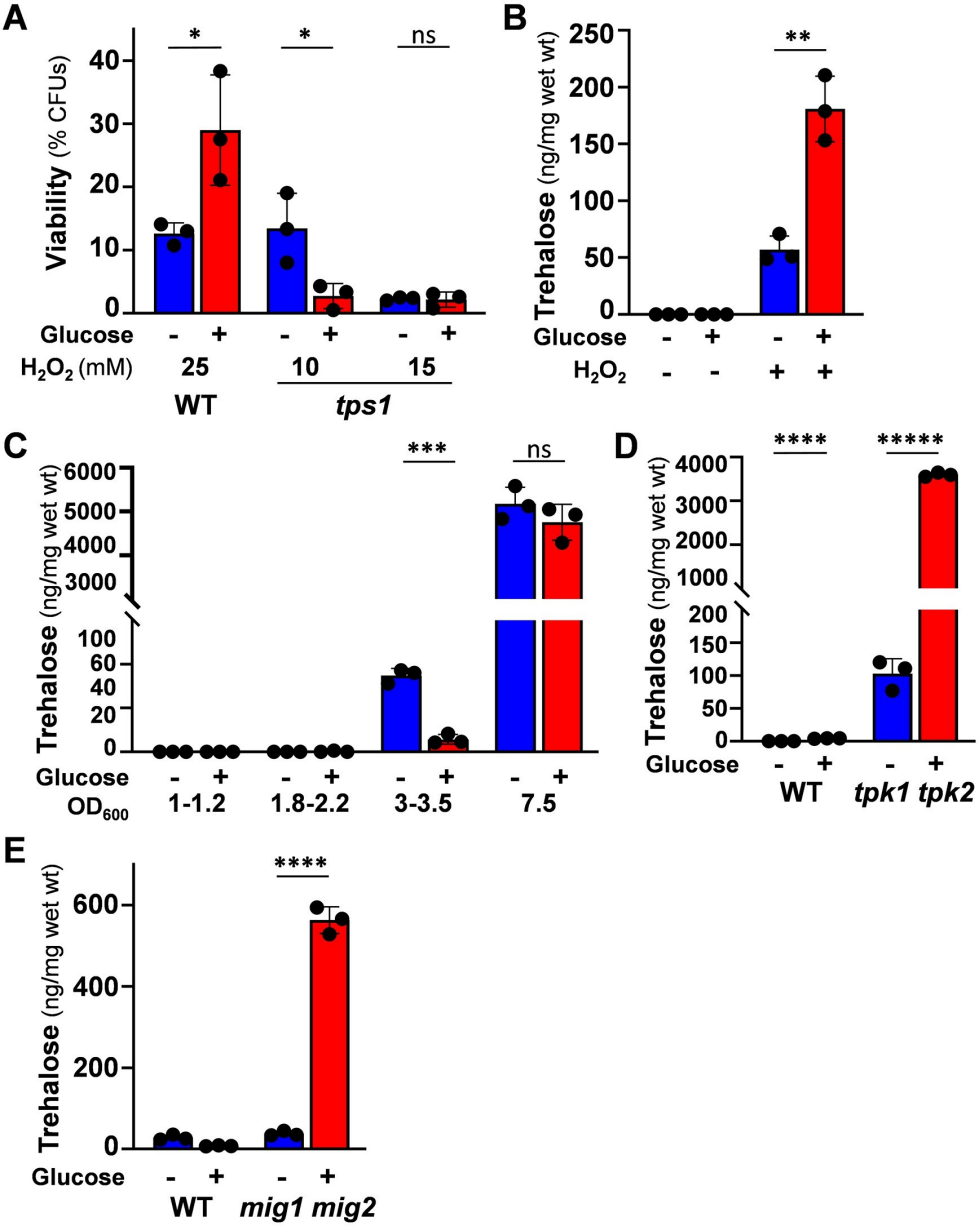
Trehalose accumulation is required for glucose-enhanced oxidative stress resistance. **(A)** The phenotype is blocked following inactivation of trehalose synthase. *C*. *albicans* strains were grown on lactate (YPL), incubated with 0 or 1% glucose for 1 h, then exposed for 1 h to H_2_O_2_ at the specified concentrations, and cell viability measured (CFUs): Ca372, CAI4+CIp10, wild type, WT; *tps1* (S1 Table). **(B)** Glucose enhances the induction of trehalose levels in response to oxidative stress. Trehalose levels were measured in *C*. *albicans* SC5314 cells grown in YPL to an OD_600_ = 1, exposed to glucose for 1h, and then treated with 0 or 25 mM H_2_O_2_ for 30 min. **(C)** Glucose does not enhance trehalose accumulation in stationary phase cells. Trehalose levels were assayed in *C*. *albicans* SC5314 cells grown in YPL to different growth phases (OD_600_) and then exposed to glucose for 1h. **(D)** Trehalose accumulation is derepressed in the absence of glucose in *C*. *albicans* cells lacking PKA. Trehalose levels were measured in *C*. *albicans* cells grown in YPL to OD_600_ = 3.0–3.5 and then exposed to glucose for 1h: SN152HLA, wild type, WT; *tpk1 tpk2*. **(E)** The inactivation of Mig1 and Mig2 derepresses trehalose accumulation in the absence of glucose. Trehalose levels were measured in *C*. *albicans* cells grown in YPL to OD_600_ = 3.0–3.5 and then exposed to glucose for 1h: *C*. *albicans* CW542, wild type, WT; *mig1 mig2*. The data in (A), (B), (C), (D) and (B) were analysed using two-way ANOVA, comparing each test (plus glucose) to the corresponding control (minus glucose). Means and standard deviations for triplicate experiments are presented: *, *p* ≤ 0.05; **, *p* ≤ 0.01; ***, *p* ≤ 0.001; ****, *p* ≤ 0.0001.

We tested this possibility previously, and apparently excluded it, using a *C*. *albicans tps1*Δ mutant [17], which lacks trehalose-6-phosphate synthase, the enzyme that catalyses the first step in trehalose synthesis. However, we discovered that this *tps1*Δ mutant is sensitive to hypoosmotic stress, and hence to the cell dilutions used in our viability assays (S1 Fig). This *tps1*Δ mutant and its wild type control were also auxotrophic for uridine (*ura3*Δ)–an issue because UDP-glucose is a substrate for trehalose synthesis. Therefore, we revisited the role of trehalose in glucose-enhanced oxidative stress resistance using prototrophic *tps1*Δ (Ca2489) and wild type control strains (Ca372: S1 Table) and an osmotic stabiliser for the cell dilutions (Materials & Methods). Lower hydrogen peroxide concentrations were used for the *C*. *albicans tps1*Δ cells because they are relatively sensitive to acute oxidative stresses [65]. In contrast to the isogenic wild type control, which displayed the normal glucose-enhanced oxidative stress resistance phenotype, the resistance of these *tps1*Δ cells to oxidative stress *decreased* in response to glucose (Fig 8A). This suggested that trehalose synthesis does promote this phenotype.

We examined the impact of glucose upon trehalose accumulation. Prior glucose exposure significantly enhanced trehalose accumulation in mid-exponential wild type *C*. *albicans* cells in response to an acute oxidative stress (Fig 8B). The unstressed control cells did not accumulate trehalose at detectable levels, suggesting that prior glucose exposure enhances the ability of *C*. *albicans* cells to rapidly synthesise trehalose in response to oxidative stress. In contrast, glucose exposure did not enhance the ability of stationary phase *C*. *albicans* cells to accumulate trehalose (Fig 8C). Therefore, the glucose enhancement of potential trehalose accumulation relates to the response to oxidative stress, not growth phase.

Given that glucose-enhanced oxidative stress resistance is compromised in *mig1*Δ *mig2*Δ and *tpk1*Δ *tpk2*Δ mutants (Figs 4E & 5E), we tested whether trehalose accumulation was also affected in these strains. Compared to the wild type controls, trehalose accumulation was derepressed in *tpk1*Δ *tpk2*Δ (Fig 8D) and *mig1*Δ *mig2*Δ cells (Fig 8E), especially following glucose exposure. Therefore, the derepression of glucose-enhanced oxidative stress resistance in these mutants correlated with the derepression of trehalose accumulation. Taken together, our data suggest that trehalose accumulation promotes glucose-enhanced oxidative stress resistance.

### Glucose-enhanced oxidative stress resistance is not observed in other yeast species

**Fig 9:**
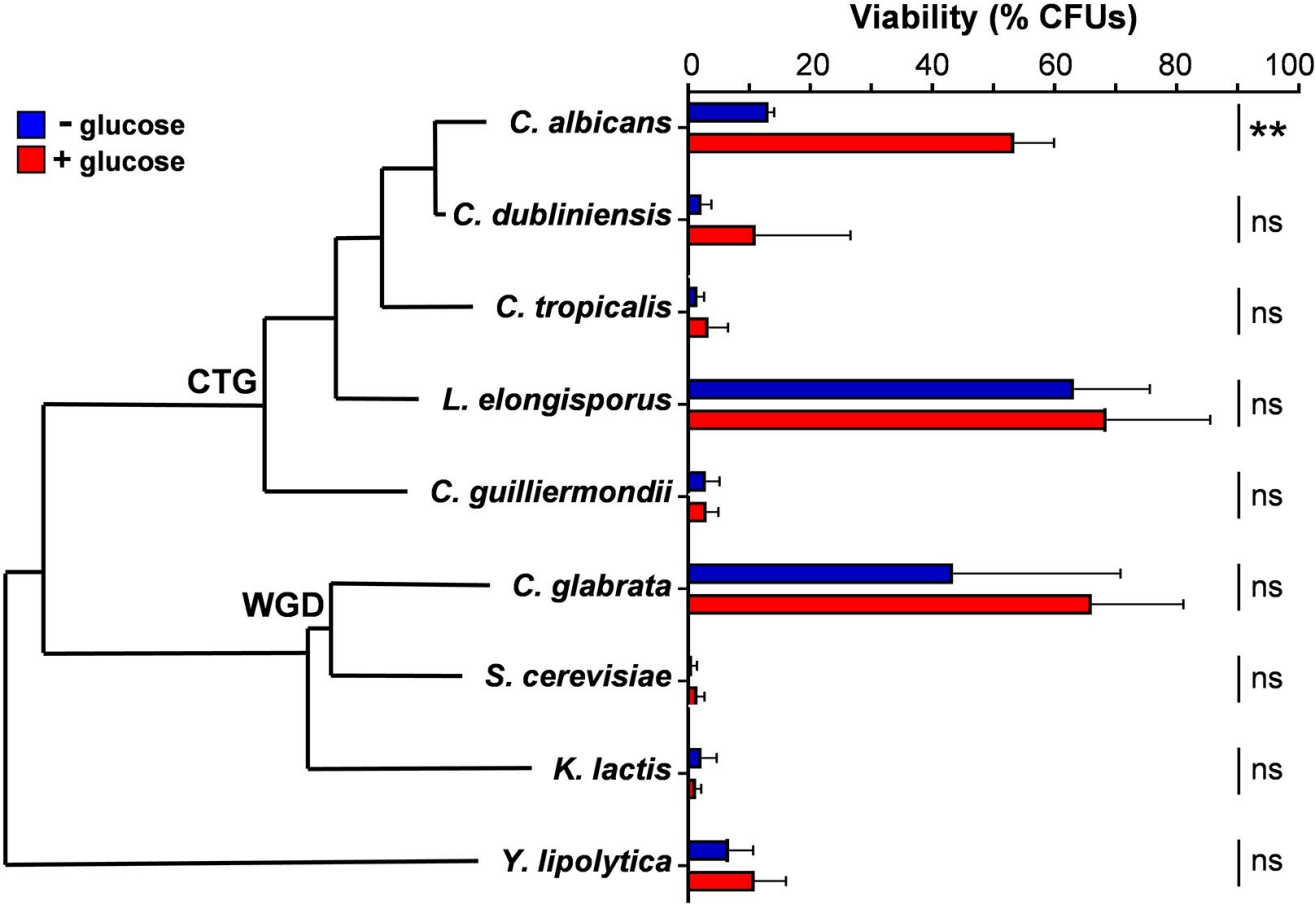
Glucose-enhanced oxidative stress resistance is not a general trait in yeast species. The phylogenetic relationships of the analysed yeast species was adapted from the tree of Saccharomycotina species published by Roetzer and co-authors [108]. Mid-exponential yeast cells grown in YPL were treated with 0 or 1% glucose for 1 h and then exposed to 50 mM H_2_O_2_ for 1 h. The yeast species were: *C*. *albicans* SC5314; *C*. *dubliniensis* IHEM20468; *C*. *tropicalis* SC S74663; *L*. *elongisporus* NRLL YB-4239; *C*. *guilliermondii BB418024*; *C*. *glabrata* AM2004/0093; *S*. *cerevisiae* BB708832; *K*. *lactis* NRRL Y-1140; *Y*. *lipolytica* CLIB122 (S1 Table). The data were analysed using two-way ANOVA, comparing each test (plus glucose) to the corresponding control (minus glucose). Means and standard deviations for triplicate experiments are presented: *, *p* ≤ 0.05; **, *p* ≤ 0.01.

Isolates from four major epidemiological clades of *C*. *albicans* (clades 1–4) have been shown to display glucose-enhanced oxidative stress resistance [17]. This phenotype was one of the first reported examples of an anticipatory protective response in a pathogenic yeast. Given that anticipatory protective responses probably reflect relatively recent evolutionary pressures [11,67], we tested whether this phenotype is observed in other pathogenic yeast species (*Candida glabrata*, *Candida tropicalis*, *Candida guilliermondii*, *Candida dubliniensis* and *Lodderomyces elongisporus* [68,69]) and other more evolutionarily divergent yeasts (*Saccharomyces cerevisiae*, *Kluyveromyces lactis*, *Yarrowia lipolytica*). As expected [70–72], these species displayed differing degrees of resistance to oxidative stress (Fig 9). Interestingly, *Candida albicans* was the only species tested that displayed significant glucose-enhanced oxidative stress resistance.

## Discussion

The fungal pathogen *C*. *albicans* responds to physiological levels of glucose by activating an oxidative stress response that protects the fungus from subsequent exposure to an acute oxidative stress [17]. We have now shown that this response is controlled by glucose signalling rather than glucose catabolism. We have also shown that the glucose-enhanced oxidative stress resistance phenotype is down-regulated by the glucose repression pathway and by cAMP-PKA signalling, and these pathways control the trehalose accumulation that underlies the phenotype. We note that potential crosstalk between the cAMP-PKA and glucose repression pathways in *C*. *albicans* might place Mig1/2 under the control of both pathways [73]. For example, in *Aspergillus nidulans*, PKA is known to regulate the Mig1 homologue, CreA [74]. Therefore, it is conceivable that the Mig1/2 mediated regulation of trehalose levels in response to glucose is regulated by cAMP-PKA signalling as well as via the glucose repression pathway.

Tps1 and trehalose accumulation contribute to the virulence of *C*. *albicans* in multiple ways. In addition to compromising stress protection, the inactivation of Tps1 or trehalose accumulation affects growth, inhibits yeast-hypha morphogenesis under certain conditions, and reduces resistance to phagocytic killing [65,66,75,76]. Our data indicate that trehalose accumulation also promotes glucose-enhanced oxidative stress resistance. Given this pleiotropy, it is not surprising that *C*. *albicans tps1* cells display significantly attenuated virulence [76].

Glucose exposure protects *C*. *albicans* against oxidative stress before this stress has been encountered, and hence this phenotype represents an anticipatory protective response [17]. We have suggested that this fungus has “learned” over evolutionary time that if cells are exposed to physiological levels of glucose, through exposure to blood, for example, they are likely to be subject to phagocytic attack shortly thereafter [11,67]. Consequently, the glucose-enhanced oxidative stress resistance phenotype might represent a form of “fungal memory” that promotes fitness *in vivo* by increasing the ability of *C*. *albicans* to counter phagocytic killing [11,67]. In this study we have now confirmed that glucose-enhanced oxidative stress resistance reduces the ability of human neutrophils to kill *C*. *albicans*, and that cells that have been exposed to glucose display increased fitness during the early stages of a systemic infection. Therefore, this anticipatory protective response has *in vivo* relevance. Interestingly, *C*. *albicans* cells that have been exposed to plasma show an even greater fitness advantage, suggesting that additional blood components might enhance this anticipatory response. Peptidoglycan might seem a potential candidate because it promotes yeast-hypha morphogenesis in *C*. *albicans* by activating cAMP-PKA signalling [77]. However, peptidoglycan is unlikely to promote glucose-enhanced oxidative stress resistance as cAMP-PKA signalling down-regulates, rather than induces, this phenotype. Therefore, other plasma components, which remain to be defined, would appear to enhance oxidative stress resistance in *C*. *albicans*.

Our examination of glucose-enhanced oxidative stress resistance in other yeasts revealed that *C*. *albicans* was the only species of those tested that displays the phenotype. Some of the species examined are environmental yeasts (*S*. *cerevisiae*, *K*. *lactis*, *Y*. *lipolytica*, for example) and some are pathogenic *Candida* species that have environmental reservoirs (*C*. *tropicalis* and *Candida guilliermondii*, for example [78,79]). Others have genomes that suggest evolution outside of the mammalian host (*C*. *glabrata*, for example [80]). Therefore, the evolutionary pressures that these fungi have faced are likely to differ significantly from *C*. *albicans*, which is a commensal of humans that is thought, despite isolations from the environment [81,82], to be obligately associated with warm-blooded animals [83–85]. The cost-benefits of glucose-enhanced oxidative stress resistance may be low in environmental niches. However, the absence of this phenotype in *C*. *dubliniensis* is striking as this species is closely related to *C*. *albicans*. (These species diverged only about 20 million years ago, whereas *S*. *cerevisiae* and *C*. *albicans* diverged about 140 million years ago [86].) *C*. *dubliniensis* is thought to be undergoing reductive evolution from *C*. *albicans* [87]. Whilst *C*. *albicans* is a common cause of both mucosal and systemic infection [88–90], *C*. *dubliniensis* is less pathogenic and is most commonly associated with mucosal infections [87,91–93]. Presumably, the cost-benefits of glucose-enhanced oxidative stress resistance are low during mucosal infection or, alternatively, this phenotype has arisen in *C*. *albicans* after its divergence from *C*. *dubliniensis*. Either way, the data support the idea that this phenotype has evolved relatively recently in response to temporally linked selective pressures that *C*. *albicans* faces during commensalism and possibly during transitions towards systemic infection. Also, the data reinforce the view that anticipatory protective responses can evolve relatively quickly, and hence that they reflect relatively recent evolutionary pressures [1,11,67].

As stated above, *C*. *albicans* displays additional anticipatory protective responses. These include candidalysin and zincophore production during hyphal development [8,9], and β-glucan masking and immune evasion in response to in-host signals [12–16]. Interestingly, these phenotypes, like glucose-enhanced oxidative stress resistance, are regulated by cAMP-PKA signalling [8,9,15,53]. Similarly, the core stress response in *S*. *cerevisiae*, another anticipatory protective response [1], is regulated by PKA signalling [94–96]. Therefore, PKA appears to be a critical regulatory hub that permits the development of anticipatory protective responses in yeasts.

## Materials and methods

### Ethics statement

Blood from healthy volunteers was collected with the informed and written consent of these donors and according to local guidelines and regulations. Our full study protocol was approved by the College Ethics Review Board of the University of Aberdeen (CERB/2016/8/1300).

Animal experiments were approved by the Animal Welfare and Ethical Review Boards at the Universities of Aberdeen and Exeter and performed in compliance with animal research ethical regulations under UK Home Office project licence number P79B6F297. BALB/c mice were purchased from Charles River and housed under specific pathogen-free conditions.

### Strain construction and growth conditions

*C*. *albicans* strains (S1 Table) were grown in 1% yeast extract, 2% bacto-peptone (YP) containing 2% glucose (YPD), 2% sodium lactate (YPL) or 2% sodium citrate (YPC) [17,97].

An isogenic set of wild type *C*. *albicans* strains, each containing a unique barcode (S2 Table), was generated by transforming *C*. *albicans* CAI4 (*ura3*Δ) with CIp10-PTET-GTW (*URA3*) plasmids from the *C*. *albicans* ORFeome project [98]. In each case, correct integration of the plasmid at the *RPS1* locus was confirmed by diagnostic PCR [99]. Similarly, the *tps1*Δ strain Ca2489 was transformed with CIp10 [99], to ensure restoration of wild type virulence phenotypes [100].

### Stress assays

*C*. *albicans* strains were grown overnight at 30°C in YPL to mid-log phase (OD_600_ = 0.5), and then subcultured into fresh YPL and re-grown to mid-log phase (OD_600_ = 0.5). Cultures were then split, and each portion received glucose at the specified concentration, human plasma from healthy volunteers (20% final concentration) or water (the control) and incubated for 60 min at 30°C. The samples were then exposed for 60 min to H_2_O_2_ at the specified concentration, after which cell viability was assayed (CFUs) relative to the corresponding unstressed control. (For viability assays with the *tps1*Δ strain, the cell dilutions were performed in 1 M sorbitol rather than phosphate buffered saline cells, because the *tps1*Δ cells were found to be hypersensitive to hypoosmotic shock (S1 Fig).) Three independent replicates were performed for each experiment.

### Catalase assays

Catalase assays were performed as described previously [101]. Mid-exponential cells were washed first with ice-cold water, and then with 50 mM sodium phosphate buffer, pH 7.5. Protein extracts were prepared, 5 μl of cell extract added to 1 ml of assay mixture (50 mM sodium phosphate buffer, pH 7.5, 0.05 mM H_2_O_2_), and the absorbance 240 nm measured every 10 seconds thereafter. Catalase activities (rates of A_240_ decline, and hence H_2_O_2_ decomposition) were normalised against total protein (μmol decomposed H_2_O_2_/min/mg protein). Assays were performed in triplicate.

### Glutathione assays

Glutathione (GSH) and glutathione disulphide (GSSG) were quantified using published procedures [59,102]. Samples (25 μl) were derivatised by adding 100 μl of 20 mM N-ethylenemaleimide, 2 mM ethylenediaminetetraacetic acid, 250 μM γ**-**glu–glu (in water:methanol 85:15) and 25 μl of 2% sulfosalicylic acid (Sigma-Aldrich), and incubating at room temperature for one hour. After derivatisation, 500 μl of water were added. Samples were then centrifuged at 13,000 rpm for 3 min, and 5 μl of the supernatant was subjected to liquid chromatography—tandem mass spectrometry using a Thermo Surveyor liquid chromatography system (150 x 2 mm Stability 100 BS-C17 column: Hichrom, Reading, UK) coupled to a TSQ Quantum triple quadrupole mass spectrometer (Thermo Fisher). Quantification was performed against freshly made standards using Xcalibur software with the following selected reaction monitoring transitions: Glu-Glu (internal standard) m/z 277–241 at 12V, GSSG m/z 613–355 at 18V. Data were normalised against total protein. Redox potential (ΔE) was calculated using the following equation: (ΔE = -178.5MV– 30.1*log*([GSH]^2^/[GSSG].*mV* [59,103,104]).

### Trehalose assays

Overnight cultures of *C*. *albicans* grown in YPL at 30°C were subcultured into 200 ml fresh YPL to an OD_600_ of 0.2 and grown for 5 hours or until they reached the indicated OD_600_. Cells were harvested, washed in 20 ml chilled PBS, and centrifuged to remove excess liquid before determining the wet weight of cell pellets. The cells were then resuspended in 300 ul Milli-Q water and boiled at 110°C for one hour. Then, 0.4 g of acid-washed 0.1 mm glass beads were added, the samples vortexed for 5 minutes in a Fastprep-24, and centrifuged at 13,000 rpm for 5 minutes. Supernatants (250 μl) were stored at -20°C before analysis. Trehalose assays were performed using the Megaenzyme Trehalose Assay Kit (Megaenzyme Inc., Bray, Ireland) on 20 μl extract according to the manufacturer’s instructions [105]. Measurements were based on three independent samples, all extracts were tested in duplicate, and controls without hexokinase and/or trehalase enzymes were included in each run. Trehalose levels were normalised against the wet weight of cell pellets.

### Neutrophil assays

Isogenic barcoded *C*. *albicans* strains (see *Strain construction*) were grown in YPL at 30°C overnight, subcultured into fresh YPL to an OD_600_ of 0.1, grown for 4 h to and OD_600_ of 0.5, and then exposed to 1% glucose, 20% human plasma, or water (the control) for one hour. Five barcoded strains were used as internal technical replicates for each of these three experimental conditions. The various, differentially adapted, barcoded strains were then harvested, washed in PBS, and pooled together in approximately equal concentrations in RPMI 1640 without glucose containing 10% foetal calf serum (Gibco). Meanwhile, human PMNs were prepared from blood donated by healthy volunteers and collected in ethylenediaminetetraacetic acid-coated vacuum phlebotomy tubes (BD Biosciences). The PMNs were prepared by density gradient centrifugation using Histopaque 1077 and Histopaque 1119 (Sigma-Aldrich) as described previously [106]. The pool of 15 barcoded *C*. *albicans* strains was then co-incubated for 3 h with human PMNs at a ratio of 10:1 (500,000 PMNs to 50,000 yeast) at 37°C under 5% CO_2_. The PMNs were then lysed for 10 min with 100 μl 0.25% SDS, mixed with 900 μl ice-cold water, and the samples incubated with 50 U of DNAse I for 15 minutes. The *C*. *albicans* cells were then harvested by centrifugation, plated onto YPD agar to recover colonies for barcode sequencing (below). Three independent experiments were performed, each with n = 5 barcodes corresponding to technical replicates for each condition.

### Animal experiments

Groups of four barcoded *C*. *albicans* strains were selected as internal replicates for each of the three experimental conditions: 0% glucose (control), 1% glucose or 20% plasma. As described above, *C*. *albicans* were grown on lactate (YPL), exposed to the relevant experimental condition for one hour, harvested and washed in PBS, and then pooled in approximately equal proportions. This pool (100 μl containing 7 x10^5^ *C*. *albicans* cells) was then injected into the lateral tail vein of eight 6–10 week-old female BALB/c mice, and the mice sacrificed after 48 hours. Fungal cells from the liver, spleen, brain and kidneys were plated onto YPD for 24 h, and pooled for barcode sequencing. Therefore, eight mice were examined, each with n = 4 barcodes as technical replicates for each condition.

### Barcode sequencing

Barcode sequencing was performed essentially as described previously [57]. Genomic DNA was prepared from pooled *C*. *albicans* populations [107], and the barcodes PCR amplified using common primers (S2 Table). The amplicons were ethanol precipitated and purified using AMPure XP beads (Beckman Coulter) using the manufacturer’s instructions. The purified amplicons were indexed with Illumina unique dual indexing primers (Illumina, Inc., San Diego, CA, USA), the libraries were pooled in equimolar concentrations (1nM) and sequenced (150 paired-end) on a Novaseq 6000 (Illumina, Inc., San Diego, CA, USA). The barseq FASTQ data files were then analysed using previously described scripts [57] that determines the total number of reads and relative abundance for each barcode relative to the total number of barcode reads. Each relative barcode abundance was then normalised against its starting concentration in the population. Means for the four or five replicate barcodes for each condition are presented.

### Statistical analyses

Statistical analyses were performed with GraphPad Prism v.8.4.1. Error bars represent standard deviations from at least three biological replicates. Significance was tested using unpaired T-test to compare two variables, or one- or two-way ANOVA with Tukey posthoc testing for multiple data sets, as specified.

## Supporting information

S1 Fig *C*. *albicans tps1* cells are sensitive to hypoosmotic shock. *C*. *albicans* cells were grown in YPL to an OD_600_ of 0.70–0.75 and then diluted into PBS or fresh YPL for 20 minutes before plating onto YPD agar: *C*. *albicans* Ca372, CAI4+CIp10, wild type, WT; tps1 (S1 Table). Each data point represents the mean for 3 technical repeats; means and standard deviations are shown for two independent replicate experiments. Statistical significance in comparison to the WT control was determined by two-way ANOVA with Dunnett multiple comparison for the same condition between strains (filled lines), and unpaired t-test to compare YPL and PBS within the same strain (dotted line): *, p ≤ 0.05; **, p ≤ 0.01.
(PDF)

S1 Table Strains used in this study. (PDF)

S2 Table Barcodes and Primers used in barcode sequencing. The isogenic set of barcoded wild type *C*. *albicans* strains (S1 Table) was generated in the parental strain *C*. *albicans* CAI4 using CIp10-PTET-GTW plasmids containing the barcodes described here. These plasmids, which are from the *C*. *albicans* ORFeome project [98], were generously provided by Professor Carol Munro.
(PDF)
