# Supplementary material for: Glucose-enhanced oxidative stress resistance—A protective anticipatory response that enhances the fitness of Candida albicans during systemic infection
Source: PLoS Pathog. 2023 Jul 10;19(7):e1011505. doi: 10.1371/journal.ppat.1011505 (PMC10358912; doi:10.1371/journal.ppat.1011505)
Supplement: S1 Fig — C. albicans cells were grown in YPL to an OD600 of 0.70–0.75 and then diluted into PBS or fresh YPL for 20 minutes before plating onto YPD agar: C. albicans Ca372, CAI4+CIp10, wild type, WT; tps1 (S1 Table). Each data point represents the mean for 3 technical repeats; means and standard deviations are shown for two independent replicate experiments. Statistical significance in comparison to the WT control was determined by two-way ANOVA with Dunnett multiple comparison for the same condition between strains (filled lines), and unpaired t-test to compare YPL and PBS within the same strain (dotted line): *, p ≤ 0.05; **, p ≤ 0.01. (PDF) [file ppat.1011505.s001.pdf]

Figure S1

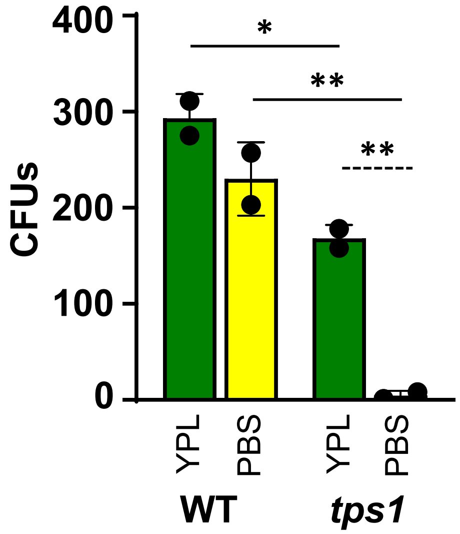

Figure S1: *tps1* cells are sensitive to hypoosmotic shock (do not survive dilution into PBS). WT = CAI4 + CIp10. All cells were grown in YPL until OD_{600} of 0.70 - 0.75. Cells were then diluted into PBS or fresh YPL for 20 minutes before plating onto YPD agar. Each data point represents the mean for 3 technical repeats; means and standard deviations are show for two independent replicate experiments. Statistical significance in comparison to the WT control was determined by two-way ANOVA with Dunnett multiple comparison for the same condition between strains (filled lines), and unpaired t-test to compare YPL and PBS within the same strain (dotted line). * $P<0.05$ ** $P<0.01$
