# Supplementary material for: Glucose-enhanced oxidative stress resistance—A protective anticipatory response that enhances the fitness of Candida albicans during systemic infection
Source: PLoS Pathog. 2023 Jul 10;19(7):e1011505. doi: 10.1371/journal.ppat.1011505 (PMC10358912; doi:10.1371/journal.ppat.1011505)
Supplement: S1 Table — (PDF) [file ppat.1011505.s002.pdf]

Table S1. Strains used in this study

Strain	Name	Parent	Genotype	Reference/Source
SC5314	SC5314	-	blood isolate	1
CAI4	CAI4	SC5314	*ura3Δ::imm434/Δura3Δ::imm434*	2
Ca372	CAI4+CIp10	CAI4	CAI4, *RPS1-CIp10 (URA3)*	3
RM1000	RM1000	CAI4	*ura3Δ::imm434/Δura3Δ::imm434, his1Δ::hisG/his1Δ::hisG*	4
Ca1674	RM1000+CIp20	RM1000	RIM1000, *RPS1-CIp20 (URA3,HIS1)*	5
BWP17	BWP17	RM1000	*ura3Δ::imm434/Δura3Δ::imm434, his1Δ::hisG/his1Δ::hisG, arg4Δ::hisG/arg4Δ::hisG*	6
Ca1206	BWP17+CIp30	BWP17	BWP17, *RPS1-CIp30 (URA3,HIS1,ARG4)*	7
SN152	SN152	SC5314	*arg4Δ/arg4Δ, leu2Δ/leu2Δ, his1Δ/his1Δ, URA3/ura3Δ::imm434, IRO1/iro1Δ::imm434*	8
CM9	*hgt4Δ*	BWP17	BWP17, *hgt4Δ::UAU1/hgt4Δ::URA3*	9
CM162	*rgt1Δ*	BWP17	BWP17, *rgt1Δ::FRT/rgt1Δ::FRT*	9
CM64	*hgt12Δ*	BWP17	BWP17, *hgt12Δ::UAU1/hgt12Δ::UAU1*	10
hgt1Δ	*hgt1Δ*	SN152	SN152, *hgt1Δ::HIS1/hgt1Δ::LEU2*	11
HGT1	*HGT1*	SN152	SN152, *hgt1Δ::HIS1/hgt1Δ::LEU2-HGT1*	11
sak1Δ	*sak1Δ*	SC5314	*sak1Δ::FRT/sak1Δ::FRT*	12
jjh85	*kis1Δ*	BWP17	BWP17, *kis1Δ::UAU1/kis1Δ::UAU1*	13
snf4Δ	*snf4Δ*	BWP17	BWP17, *snf4Δ::UAU1/snf4Δ::UAU1*	Aaron Mitchell
mig1Δ	*mig1Δ*	SN152	SN152, *mig1Δ::CdHIS1/mig1Δ::CmLEU2*	14
mig2Δ	*mig2Δ*	SN152	SN152, *mig2Δ::CdARG4/mig2Δ::CdARG4*	14
mig1Δ mig2Δ	*mig1Δ mig2Δ*	SN152	SN152, *mig1Δ::CdHIS1/mig1Δ::CmLEU2, mig2Δ::CdARG4/mig2Δ::CdARG4*	14
ras1-2/ras1-3	*ras1Δ*	CAI4	CAI4, *ras1Δ::hisG/ras1Δ::hph-URA3-hph*	15
LR2	*gpr1Δ*	CAI4	CAI4, *gpr1Δ::hisG/gpr1Δ::hisG-URA3-hisG*	16
NM6	*gpa2Δ*	CAI4	CAI4, *gpa2Δ::hisG/gpa2Δ::hisG-URA3-hisG*	16
NM23	*gpr1Δ gpa2Δ*	CAI4	CAI4, *gpa2Δ::hisG/gpa2Δ::hisG, gpr1Δ::hisG/gpr1Δ::hisG-URA3-hisG*	16
CR323	*cyr1Δ* (*cdc35Δ*)	CAI4	CAI4, *cdc35Δ::hisG/cdc35Δ::hisG, pVEC-URA3*	17
WH2-3U	*pde2Δ*	CAI4	CAI4, *pde2Δ::hisG/pde2Δ::hisG-URA3-hisG*	18
tpk1Δ	*tpk1Δ*	SN152	SN152, *tpk1Δ::HIS1/ tpk1Δ::ARG4*	19
tpk2Δ	*tpk2Δ*	SN152	SN152, *tpk2Δ::HIS1/ tpk2Δ::ARG4*	19
tpk1Δ tpk2Δ	*tpk1Δ tpk2Δ*	SN152	SN152, *tpk1Δ::LEU2/ tpk1Δ::FRT, tpk2Δ::HIS1/ tpk2Δ::ARG4*	19
Ca1862	*CTA1/cta1Δ*	RM1000	RIM1000, *cta1Δ::loxP-URA3-loxP/CTA1*	This study
Ca1864	*cta1Δ/cta1Δ*	RM1000	RIM1000, *cta1Δ:: loxP-URA3-loxP/cta1Δ::HIS1*	This study
Ca2015	*gsh2Δ*	RM1000	RM1000, *gsh2Δ::loxP/gsh2Δ::loxP*	20
C20	*tps1Δ* (*ura3$^-$*)	CAI4	CAI4, *tps1Δ::hisG/tps1Δ::hisG*	21
Ca2489	*tps1Δ*	CAI4	CAI4, *tps1Δ::hisG/tps1Δ::hisG, RPS1-CIp10 (URA3)*	This study

**BARCODED *C. ALBICANS* STRAINS**			
Genotype: CAI4, *RPS1-CIp10-pTET-GW (URA3)*			
**Strain**	**Barcode**	**Barcode sequence** (common 5’ sequence-**unique barcode**-common 3’ sequence)	**Source**
Ca 2247	BC_A03	CGGTGTCGGTCTCGTAG**TACAATAAAGGGCAGGTGCAACCAT**AGAGACCTCGTGGACATC	This study
Ca 2248	BC_A08	CGGTGTCGGTCTCGTAG**CACAGACGACGTAACTTTTATAGGC**AGAGACCTCGTGGACATC	This study
Ca 2249	BC_A10	CGGTGTCGGTCTCGTAG**TGACCAAGCCTGAATAGCGTCATAA**AGAGACCTCGTGGACATC	This study
Ca 2250	BC_A11	CGGTGTCGGTCTCGTAG**CGTGTATTAGAGTAATCGCATCTAG**AGAGACCTCGTGGACATC	This study
Ca 2251	BC_A12	CGGTGTCGGTCTCGTAG**ACGAAGCGTGAAACGTATCGAACGC**AGAGACCTCGTGGACATC	This study
Ca 2252	BC_B04	CGGTGTCGGTCTCGTAG**TAGCGTTAGCGGCCACGGAACATCA**AGAGACCTCGTGGACATC	This study
Ca 2253	BC_B05	CGGTGTCGGTCTCGTAG**TTAATTGCGGGCAGCCACCGTGAAT**AGAGACCTCGTGGACATC	This study
Ca 2254	BC_B07	CGGTGTCGGTCTCGTAG**TCCTCTCGGGAGGCCAATAGAAACG**AGAGACCTCGTGGACATC	This study
Ca 2255	BC_B08	CGGTGTCGGTCTCGTAG**TACGCTTGACCCGTGTAGCTGTCAG**AGAGACCTCGTGGACATC	This study
Ca 2256	BC_B11	CGGTGTCGGTCTCGTAG**TGGTTAACACGGAACCTGAGATCCC**AGAGACCTCGTGGACATC	This study
Ca 2257	BC_C10	CGGTGTCGGTCTCGTAG**TAGCACACCTAGCGTGAACCGAGCC**AGAGACCTCGTGGACATC	This study
Ca 2258	BC_C11	CGGTGTCGGTCTCGTAG**TCTAATAACCCCGGATAGGCATGCC**AGAGACCTCGTGGACATC	This study
Ca 2259	BC_C12	CGGTGTCGGTCTCGTAG**TTTAAGCATACTCCCATCCGTGCGC**AGAGACCTCGTGGACATC	This study
Ca 2260	BC_D01	CGGTGTCGGTCTCGTAG**TATCTGAGTGTTTGCGGGCTATGCA**AGAGACCTCTTGGACATC	This study
Ca 2261	BC_D02	CGGTGTCGGTCTCGTAG**TAGTTAGGGCGGCCGAAGTTATCCA**AGAGACCTCGTGGACATC	This study

**YEAST SPECIES**			
**Species**	**Strain**	**Information**	**Reference/Source**
C. albicans	SC5314	Clinical isolate	1
C. dubliniensis	IHEM20468	Clinical isolate	D. MacCallum
C. tropicalis	SC S74663	Clinical isolate	D. MacCallum
L. elongisporus	NRLL YB-4239	*L. elongisporus* often isolated from the environment	J. Heitman
C. guilliermondii	BB418024	Clinical isolate	D. MacCallum
C. glabrata	AM2004/0093	Clinical isolate	D. MacCallum
S. cerevisiae	BB708832	Clinical isolate	D. MacCallum
K. lactis	NRRL Y-1140	Isolated from milk	LGC-Promochem (Teddington, Middlesex, UK)
Y. lipolytica	CLIB122	*Y. lipolytica* often isolated from foods	Collection de Levures d’Interet Biotech

References

1. Gillum AM *et al.* (1984) Isolation of the *Candida albicans* gene for orotidine-5'-phosphate decarboxylase by complementation of *S. cerevisiae ura3* and *E. coli pyrF* mutations. *Molec. Gen. Genet*. 198, 179-182.
2. Fonzi WA & Irwin MY (1993) Isogenic strain construction and gene mapping in Candida albicans. *Genetics* 134, 717-728.
3. Murad AMA *et al.* (2000) CIp10, an efficient and convenient integrating vector for *Candida albicans*. *Yeast,* 16, 325-327
4. Negredo A *et al.* (1997) Cloning, analysis and one-step disruption of the ARG5,6 gene of *Candida albicans*. *Microbiology* 143, 297-302.
5. Smith DA *et al.* (2004) A conserved stress-activated protein kinase regulates a core stress response in the human pathogen *Candida albicans*. *Molec. Biol. Cell* 15, 4179-4190.
6. Wilson RB *et al.* (1999) Rapid hypothesis testing with *Candida albicans* through gene disruption with short homology regions. *J Bacteriol* 181, 1868-1874.
7. Dennison PM *et al*. (2005) Gene disruption in *Candida albicans* using a synthetic, codon-optimised Cre-loxP system. *Fungal Genet. Biol.* 42, 737-748.
8. Noble SM & Johnson AD (2005) Strains and strategies for large-scale gene deletion studies of the diploid human fungal pathogen *Candida albicans*. *Eukaryot Cell*. 4, 298-309.
9. Sexton JA *et al*. (2007) Regulation of sugar transport and metabolism by the *Candida albicans* Rgt1 transcriptional repressor. *Yeast*, 24, 847–860.
10. Brown *et al.* (2006) A glucose sensor in *Candida albicans*. *Eukaryotic Cell* 5: 1726–1737.
11. Kenno S *et al*. (2019) *Candida albicans* Factor H Binding Molecule Hgt1p – A low glucose-induced transmembrane protein is trafficked to the cell wall and impairs phagocytosis and killing by human neutrophils. *Frontiers Microbiol* 9, 3319.
12. Ramirez-Zavala *et al.* (2017) The Snf1-activating kinase Sak1 is a key regulator of metabolic adaptation and in vivo fitness of *Candida albicans*. *Molec Microbiol* 104, 989-1007.
13. Blankenship JR *et al.* (2010) An extensive circuitry for cell wall regulation in *Candida albicans*. *PLoS Pathog* 6: e1000752.
14. Lagree K *et al.* (2020) Roles of *Candida albicans* Mig1 and Mig2 in glucose repression, pathogenicity traits, and SNF1 essentiality. *PLOS Genet* 16, e1008582.
15. Feng Q *et al*. (1999) Ras signaling is required for serum-induced hyphal differentiation in *Candida albicans*. *J Bacteriol* 181**,** 6339-6346.
16. Maidan MM *et al*. (2005) The G protein-coupled receptor Gpr1 and the Galpha protein Gpa2 act through the cAMP-protein kinase A pathway to induce morphogenesis in *Candida albicans*. *Molec Biol Cell*. 16, 971-1986.
17. Rocha CRC *et al*. (2001) Signaling through adenylyl cyclase is essential for hyphal growth and virulence in the pathogenic fungus *Candida albicans*. *Molec Biol Cell* 12, 3631-3643.
18. Jung WH & Stateva LI (2003) The cAMP phosphodiesterase encoded by *CaPDE2* is required for hyphal development in *Candida albicans*. *Microbiology* 149, 2961-2976.
19. Cao C *et al.* (2017) Global regulatory roles of the cAMP/PKA pathway revealed by phenotypic, transcriptomic and phosphoproteomic analyses in a null mutant of the PKA catalytic subunit in *Candida albicans*. *Molec Microbiol* 105, 46-64.
20. Shahana S *et al*. (2014) New *Clox* systems for rapid and efficient gene disruption in *Candida albicans. PLoS ONE* 9*,* e100390.
21. Zaragoza O *et al*. (1998). Disruption of the *Candida albicans TPS1* gene encoding trehalose-6- phosphate synthase impairs formation of hyphae and decreases infectivity. *J Bacteriol* 180, 3809–3815.
