# Supplementary material for: Glucose-enhanced oxidative stress resistance—A protective anticipatory response that enhances the fitness of Candida albicans during systemic infection
Source: PLoS Pathog. 2023 Jul 10;19(7):e1011505. doi: 10.1371/journal.ppat.1011505 (PMC10358912; doi:10.1371/journal.ppat.1011505)
Supplement: S2 Table — The isogenic set of barcoded wild type C. albicans strains (S1 Table) was generated in the parental strain C. albicans CAI4 using CIp10-PTET-GTW plasmids containing the barcodes described here. These plasmids, which are from the C. albicans ORFeome project [98], were generously provided by Professor Carol Munro. (PDF) [file ppat.1011505.s003.pdf]

Table S2. Barcodes and Primers used in barcode sequencing.

Barcode ID	Sequence (5' -> 3')
A03	CGGTGTCGGTCTCGTAG**TACAATAAAGGGCAGGTGCAACCAT**AGAGACCTCGTGGACATC
A10	CGGTGTCGGTCTCGTAG**TGACCAAGCCTGAATAGCGTCATAA**AGAGACCTCGTGGACATC
A12	CGGTGTCGGTCTCGTAG**ACGAAGCGTGAAACGTATCGAACGC**AGAGACCTCGTGGACATC
B04	CGGTGTCGGTCTCGTAG**TAGCGTTAGCGGCCACGGAACATCA**AGAGACCTCGTGGACATC
B05	CGGTGTCGGTCTCGTAG**TTAATTGCGGGCAGCCACCGTGAAT**AGAGACCTCGTGGACATC
B07	CGGTGTCGGTCTCGTAG**TCCTCTCGGGAGGCCAATAGAAACG**AGAGACCTCGTGGACATC
C12	CGGTGTCGGTCTCGTAG**TTTAAGCATACTCCCATCCGTGCGC**AGAGACCTCGTGGACATC
D02	CGGTGTCGGTCTCGTAG**TAGTTAGGGCGGCCGAAGTTATCCA**AGAGACCTCGTGGACATC
A08	CGGTGTCGGTCTCGTAG**CACAGACGACGTAACTTTTATAGGC**AGAGACCTCGTGGACATC
A11	CGGTGTCGGTCTCGTAG**CGTGTATTAGAGTAATCGCATCTAG**AGAGACCTCGTGGACATC
B08	CGGTGTCGGTCTCGTAG**TACGCTTGACCCGTGTAGCTGTCAG**AGAGACCTCGTGGACATC
B11	CGGTGTCGGTCTCGTAG**TGGTTAACACGGAACCTGAGATCCC**AGAGACCTCGTGGACATC
C10	CGGTGTCGGTCTCGTAG**TAGCACACCTAGCGTGAACCGAGCC**AGAGACCTCGTGGACATC
C11	CGGTGTCGGTCTCGTAG**TCTAATAACCCCGGATAGGCATGCC**AGAGACCTCGTGGACATC
D01	CGGTGTCGGTCTCGTAG**TATCTGAGTGTTTGCGGGCTATGCA**AGAGACCTCTTGGACATC
**Barcode amplification primers**	**Sequence (5' -> 3')**
seqBC_F	TCGTCGGCAGCGTCAGATGTGTATAAGAGACAGCGCTCTTCCGATCTCCTAGG
seqBC_R	GTCTCGTGGGCTCGGAGATGTGTATAAGAGACAGGATAGCCGCGCTGCTAGC

Table S2: Barcodes and Primers used in barcode sequencing. The isogenic set of barcoded wild type *C. albicans* strains was generated in the parental strain *C. albicans* CAI4 using CIp10-PTET-GTW plasmids containing the barcodes described here. These plasmids, which are from the *C. albicans* ORFeome project [1,2], were generously provided by Professor Carol Munro.

References

1. Cabral V, Znaidi S, Walker LA, Martin-Yken H, Dague E, Legrand M, et al. Targeted changes of the cell wall proteome influence Candida albicans ability to form single- and multi-strain biofilms. PLoS Pathog. 2014;10: e1004542.
2. Chauvel M, Bachellier-Bassi S, Guérout AM, Lee KK, Maufrais C, Permal E, et al. High-throughput functional profiling of the human fungal pathogen *Candida albicans* genome. Res Microbiol. 2023; 174: 104025.
